# Validation of cardiac diffusion tensor imaging sequences: A multicentre test–retest phantom study

**DOI:** 10.1002/nbm.4685

**Published:** 2022-02-08

**Authors:** Irvin Teh, William A. Romero R., Jordan Boyle, Jaume Coll‐Font, Erica Dall'Armellina, Daniel B. Ennis, Pedro F. Ferreira, Prateek Kalra, Arunark Kolipaka, Sebastian Kozerke, David Lohr, François‐Pierre Mongeon, Kévin Moulin, Christopher Nguyen, Sonia Nielles‐Vallespin, Brian Raterman, Laura M. Schreiber, Andrew D. Scott, David E. Sosnovik, Christian T. Stoeck, Cyril Tous, Elizabeth M. Tunnicliffe, Andreas M. Weng, Pierre Croisille, Magalie Viallon, Jürgen E. Schneider

**Affiliations:** ^1^ Leeds Institute of Cardiovascular and Metabolic Medicine University of Leeds Leeds UK; ^2^ Univ Lyon, INSA‐Lyon, Université Claude Bernard Lyon 1 UJM‐Saint Etienne, CNRS, Inserm, CREATIS UMR 5220, U1294, F‐42023 Saint Etienne France; ^3^ School of Mechanical Engineering University of Leeds Leeds UK; ^4^ Cardiovascular Research Center and A. A. Martinos Center for Biomedical Imaging Massachusetts General Hospital and Harvard Medical School Boston Massachusetts USA; ^5^ Division of Radiology VA Palo Alto Health Care System Palo Alto California USA; ^6^ Department of Radiology Stanford University Stanford California USA; ^7^ Cardiovascular Magnetic Resonance Unit The Royal Brompton and Harefield NHS Foundation Trust London UK; ^8^ National Heart and Lung Institute Imperial College London London UK; ^9^ Department of Radiology The Ohio State University Wexner Medical Center Columbus Ohio USA; ^10^ Institute for Biomedical Engineering University and ETH Zurich Zurich Switzerland; ^11^ Department of Cardiovascular Imaging Comprehensive Heart Failure Center Würzburg Germany; ^12^ Division of Non‐invasive Cardiology Montreal Heart Institute Montreal Canada; ^13^ Department of Radiology, Radiation‐Oncology and Nuclear Medicine and Institute of Biomedical Engineering Université de Montréal Montréal Canada; ^14^ Radcliffe Department of Medicine University of Oxford Oxford UK; ^15^ Oxford NIHR Biomedical Research Centre Oxford UK; ^16^ Department of Diagnostic and Interventional Radiology University Hospital Würzburg Würzburg Germany

**Keywords:** cardiac DTI, isotropic phantom, multicentre, polyvinylpyrrolidone, pulse sequence validation, reproducibility

## Abstract

Cardiac diffusion tensor imaging (DTI) is an emerging technique for the in vivo characterisation of myocardial microstructure, and there is a growing need for its validation and standardisation. We sought to establish the accuracy, precision, repeatability and reproducibility of state‐of‐the‐art pulse sequences for cardiac DTI among 10 centres internationally. Phantoms comprising 0%–20% polyvinylpyrrolidone (PVP) were scanned with DTI using a product pulsed gradient spin echo (PGSE; N = 10 sites) sequence, and a custom motion‐compensated spin echo (SE; N = 5) or stimulated echo acquisition mode (STEAM; N = 5) sequence suitable for cardiac DTI in vivo. A second identical scan was performed 1–9 days later, and the data were analysed centrally. The average mean diffusivities (MDs) in 0% PVP were (1.124, 1.130, 1.113) x 10^−3^ mm^2^/s for PGSE, SE and STEAM, respectively, and accurate to within 1.5% of reference data from the literature. The coefficients of variation in MDs across sites were 2.6%, 3.1% and 2.1% for PGSE, SE and STEAM, respectively, and were similar to previous studies using only PGSE. Reproducibility in MD was excellent, with mean differences in PGSE, SE and STEAM of (0.3 ± 2.3, 0.24 ± 0.95, 0.52 ± 0.58) x 10^−5^ mm^2^/s (mean ± 1.96 SD). We show that custom sequences for cardiac DTI provide accurate, precise, repeatable and reproducible measurements. Further work in anisotropic and/or deforming phantoms is warranted.

## Graphical abstract

There is a growing need for validation of pulse sequences for cardiac diffusion tensor imaging (DTI). We show, in a multicentre study involving 10 sites, that custom sequences for cardiac DTI based on motion‐compensated spin echo and stimulated echo acquisition mode provide accurate, precise and reproducible measurements.

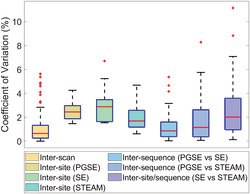

**Abbreviations used**
ADC: apparent diffusion coefficient
CODE: convex optimised diffusion encoding
CV: coefficient of variation
DTI: diffusion tensor imaging
DW: diffusion‐weighted
DWI: diffusion‐weighted imaging
EPI: echo planar imaging
FA: fractional anisotropy
HCM: hypertrophic cardiomyopathy
M2B1resist: M2SE with improved robustness to B_1_ inhomogeneities
M2SE: second‐order motion‐compensated spin echo
MD: mean diffusivity
MODE: motion‐compensated optimised diffusion encoding
PGSE: pulsed gradient spin echo
PVP: polyvinylpyrrolidone
RMSD: root mean squared difference
ROI: region of interest
SD: standard deviation
SE: spin echo
SNR: signal‐to‐noise ratio
STEAM: stimulated echo acquisition mode

## 1 INTRODUCTION

Diffusion tensor imaging (DTI) is an emerging noninvasive and contrast agent‐free method for the characterisation of cardiac microstructure. It provides measurements, such as mean diffusivity (MD) and fractional anisotropy (FA), that are sensitive to the diffusion of water molecules, and therefore local tissue structure. Increased MD, for example, corresponded well to late gadolinium‐enhanced segments in myocardial infarction,^1^, ^2^ while decreased FA can reflect cardiomyocyte disarray and increased extracellular volume in hypertrophic cardiomyopathy (HCM).^3^ Changes in MD and FA have also been associated with a range of conditions including myocardial infarction,^4^, ^5^ hypertrophy,^6^ athlete's heart,^7^ fibrosis,^8^ amyloidosis^9^ and dilated cardiomyopathy.^10^, ^11^

Despite recent advances, several major challenges need to be addressed to facilitate integration of DTI in clinical routine. The first is the uncertainty in choice of pulse sequence. As a result of cardiac motion, methods for motion compensation have had to be developed. These can be classed into two broad approaches, both employing cardiac triggering for synchronising to the cardiac cycle, and a single‐shot echo planar imaging (EPI) readout for reduced motion sensitivity. The first method is based on stimulated echo acquisition mode (STEAM)^12^ with monopolar^13^ diffusion gradient waveforms. The second method is based on spin echo (SE) with motion‐compensated diffusion gradient waveforms.^14^ The most widely used SE implementation involves up to second‐order motion compensation, rendering the sequence insensitive to constant velocity and acceleration. Second‐order motion‐compensated spin echo (M2SE) can be achieved with a range of methods, including asymmetric bipolar waveforms,^15^ symmetric tripolar waveforms with improved robustness to B_1_ inhomogeneities (M2B1resist)^16^ and numerically optimised waveforms convex optimised diffusion encoding (CODE)^17^ and motion‐compensated optimised diffusion encoding (MODE)^18^ with reduced echo times. Two studies compared the performance of STEAM and M2SE. One reported more than twofold higher signal‐to‐noise ratio (SNR) efficiency in M2SE compared with STEAM,^19^ while the other, performed on a system with standard clinical gradients, observed that STEAM was more robust over a range of cardiac phases.^20^

The second challenge is the known sensitivity of diffusion MRI, not just to tissue properties, but also to acquisition parameters. Fitting of a tensor to diffusion‐weighted (DW) data ignores non‐Gaussian diffusion effects^21^ stemming from the multiple compartments and barriers present within the complex cell microenvironment, leading to measures of apparent diffusivity that are sensitive to parameters such as pulse sequences,^19^, ^20^ diffusion times^22^ and b‐value.^23^ Furthermore, DTI relies on low SNR DW images, leading to errors in measured parameters,^24^, ^25^ and sensitivity to image resolution and SNR.^26^ The low SNR necessitates larger voxel sizes that enhance partial volume and residual motion artefacts,^27^ while the rapid switching of diffusion gradient waveforms may enhance eddy current effects.^27^ Additionally, early STEAM studies erroneously ascribed b‐values of 0 s/mm^2^ to the non‐DW data during the reconstruction, leading to bias in MD, even within one sequence type.^28^, ^29^ Consequently, studies in the myocardium of healthy volunteers have reported a wide range of MD (0.87 x 10^−3^ to 1.72 x 10^−3^ mm^2^/s) and FA (0.29 to 0.61).^19^, ^20^, ^30^, ^31^ This hampers comparison of data between sites and studies, and a standardised protocol is needed for comparison of absolute values.

The third challenge is the need to establish reproducibility of DTI parameters within and across sites. One intrasite study in healthy volunteers using a STEAM‐EPI sequence showed no significant differences in myocardial MD and FA acquired at two time points.^28^ Another single‐site study in patients with HCM^32^ reported coefficients of variation (CVs) of myocardial MD and FA across two time points of 19% and 7.2%, respectively. Intersite reproducibility is typically measured by scanning standardised subjects and substrates, such as travelling volunteers and phantoms, respectively, at multiple sites. Such studies have been performed most widely in the context of brain imaging, and have reported good reproducibility in MD and FA across up to 11 sites, with intersite CV_MD_ in brain ranging from 1.6% to 5.4% and CV_FA_ ranging from 2.0% to 4.5%.^33^, ^34^, ^35^ One cardiac DTI study involving healthy volunteers at two sites reported myocardial CV_MD_ and CV_FA_ of up to 7% and 6%, respectively.^29^

While volunteers are key to assessing the real‐world performance of DTI, phantoms are more cost‐effective for larger scale studies, offer longer term stability and allow for customisation of features for isolation of sources of variation in the data. Isotropic phantoms, for example, ice water^33^ or aqueous solutions of polyvinylpyrrolidone (PVP)^34^, ^36^, ^37^ or nickel chloride and sodium chloride,^35^ provide a homogeneous substrate with known MD, while biomimetic phantoms simulating cardiac microstructure^38^ additionally permit reproducible measurements of FA. While such anisotropic phantoms could facilitate more realistic evaluation of anisotropy, these remain generally unavailable. Moreover, isotropic phantoms have a unique advantage, in that there exist gold standard reference measurements of diffusivity in the case of water, and corroborating MD values in the literature in the case of PVP.

In this multicentre study, we sought to establish the accuracy, precision, repeatability and reproducibility of state‐of‐the‐art pulse sequences for cardiac DTI among 10 centres internationally. We employed a standardized, custom‐built, temperature‐controlled PVP phantom to permit baseline evaluation of MD and FA, as well as intrasite and intersite reproducibility in a homogeneous, isotropic substrate with known diffusivity, and acquired data using a product noncardiac DTI pulse sequence to serve as a reference.

## 2 MATERIALS AND METHODS

Ten sites participated in the study (one Philips 1.5‐T scanner, nine Siemens 3‐T scanners). Phantoms were produced at a single site and shipped to participating sites. Data were acquired using a standardised protocol prescribing both product and custom DTI sequences. A second scan was performed from 1–9 days following the first scan. Data were uploaded to a central server, and analysed by a single site using a standardised processing pipeline.

### 2.1 Phantom preparation

**FIGURE 1:**
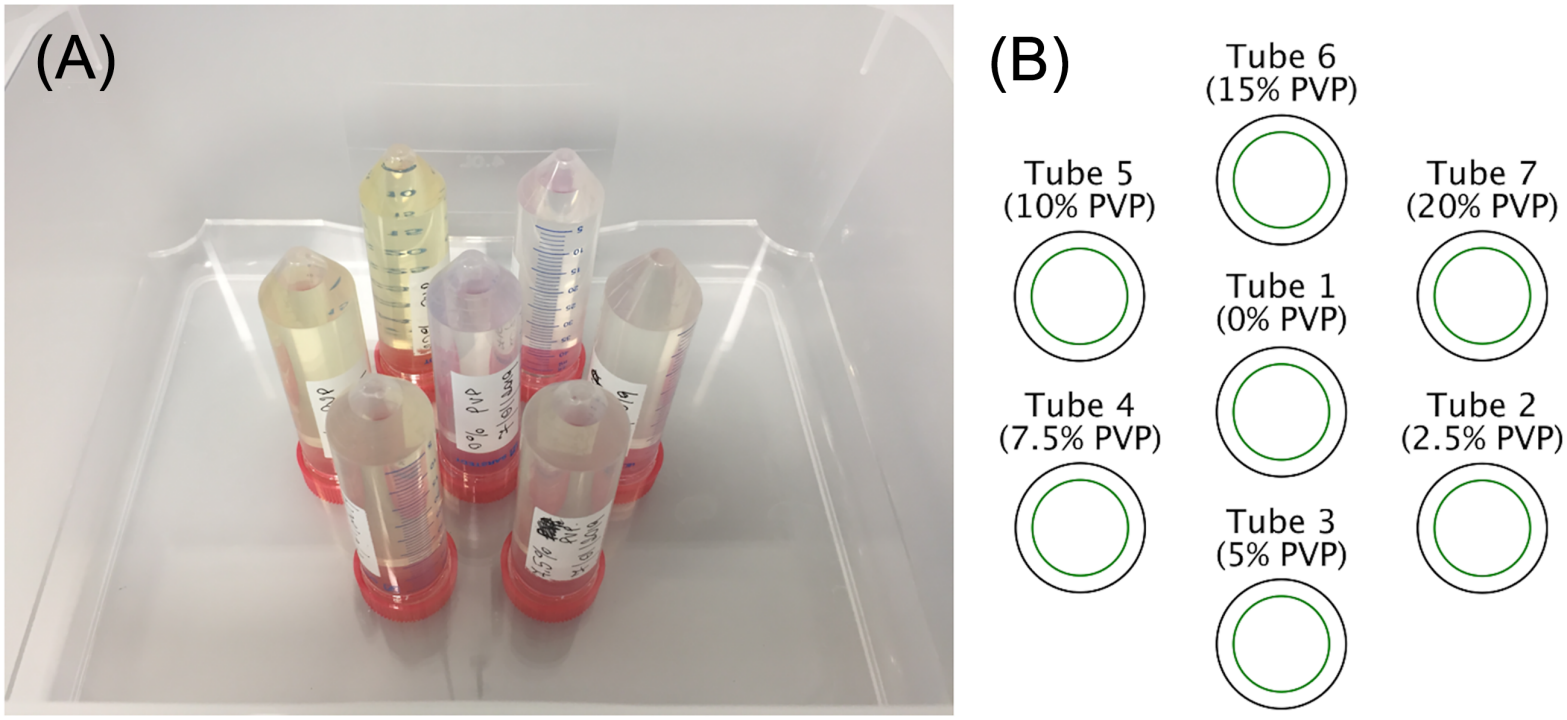
Phantom design. (A) Photograph of phantom prior to filling with ice‐water bath. (B) Key for identifying tubes with different concentrations of polyvinylpyrrolidone (PVP)

The phantom comprised 7 x 50‐ml Falcon tubes filled with PVP (Sigma Aldrich, Dorset, UK) in distilled water in concentrations ranging from 0% to 20%. The tubes were positioned vertically in an outer container using a laser‐cut plastic holder. Prior to imaging, the tubes were immersed in an ice‐water bath and given sufficient time to equilibrate to 0°C (Figure 1). The phantoms were placed at the isocentre in identical orientation.

### 2.2 Data acquisition

**FIGURE 2:**
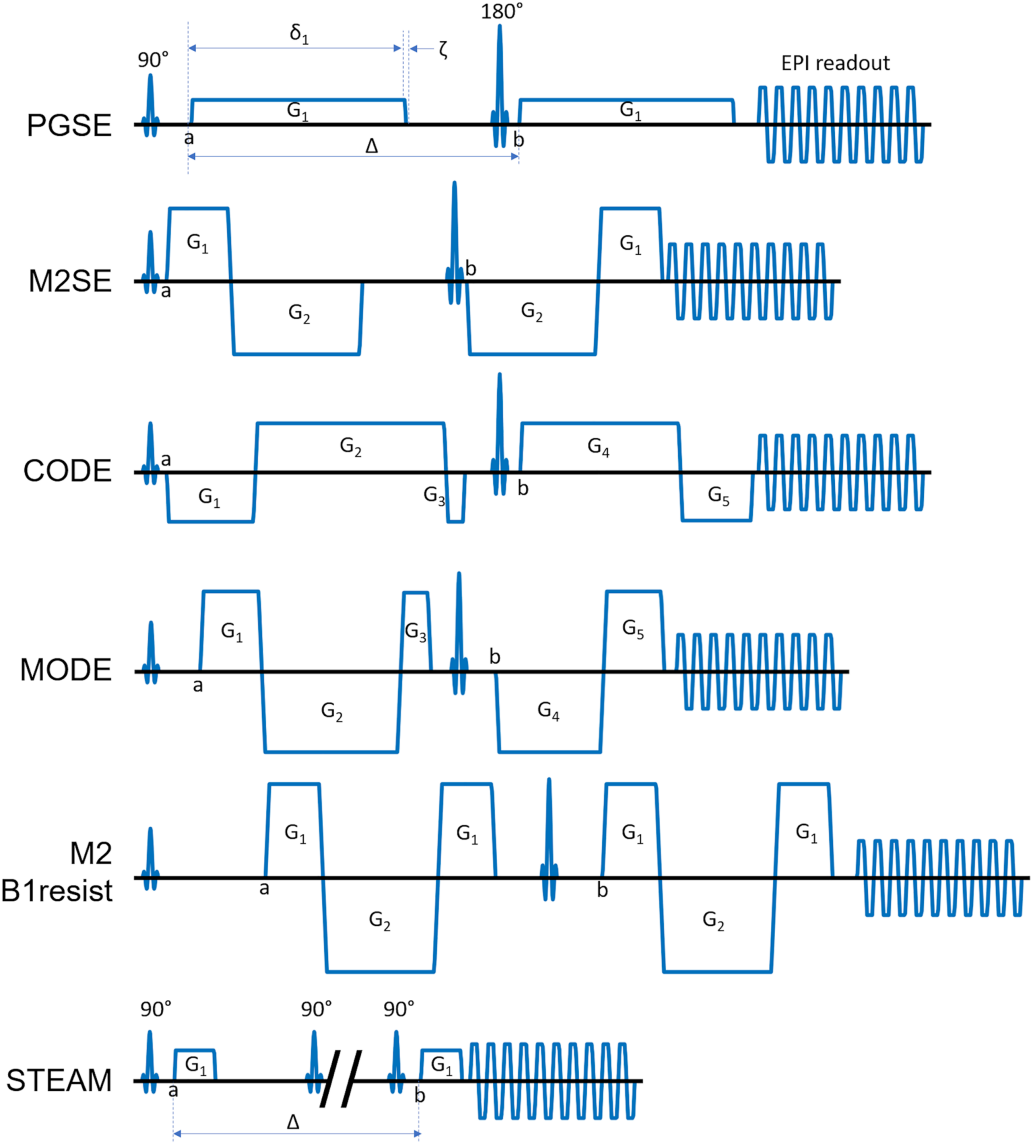
Schematic pulse sequence diagrams describing the diffusion waveforms used. These include the pulsed gradient spin echo (PGSE) product sequence and custom sequences as follows: motion‐compensated spin echo (M2SE), convex optimised diffusion encoding (CODE), motion‐compensated optimised diffusion encoding (MODE), motion‐compensated symmetric spin echo (M2B1resist) and stimulated echo acquisition mode (STEAM). All custom spin echo sequences were motion‐compensated up to the second order, and all sequences used a single‐shot echo planar imaging readout. For clarity, preparation pulses, navigators, crushers and spoilers are not shown. Gradient waveform parameters, including diffusion gradient duration (δ_n_) for each unique gradient lobe, time (b –a), slew time (ζ) and diffusion time (Δ) where applicable, are given in Table 1. δ_n_ was measured from the start of each unique gradient lobe (G_n_) to the end of its plateau. Pulse sequences are not shown to scale

To verify consistent temperature, a fast diffusion‐weighted imaging (DWI) scout scan was performed using the product sequence (i.e. pulsed gradient spin echo [PGSE] with single‐shot EPI readout). The mean apparent diffusion coefficient (ADC) was measured in a region of interest (ROI) in the central tube. The scout scan was repeated at 10‐min intervals, until the mean ADCs across two consecutive scans were within 3% agreement. Sites performed experiments using the product PGSE sequence^13^ and a custom DTI sequence of their choice optimised for cardiac‐specific applications. The scan parameters for the product sequence were TR/TE = 3000/85 ms, coronal view, field of view (FOV) = 300 × 230 mm, in‐plane resolution = 2.5 mm, slice thickness = 8 mm, gap = 8 mm, slices = 3, bandwidth ~ 3000 Hz, parallel imaging acceleration = 2x, ECG‐triggered with simulated heart rate = 60 beats per min. Specified parameters such as TE and resolution were fairly conservative, to ensure that all sites could meet the sequence specifications in the presence of different hardware capabilities. To ensure consistency in diffusion schemes across different scanners, the following diffusion schemes were specified: (i) DWI scout scan: non‐DW images = 1, diffusion vectors (3) = [1 0 0; 0 1 0; 0 0 1], b = 450 s/mm^2^, repetitions = 3, acquisition time ~36 s; (ii) DTI scan: non‐DW images = 1, diffusion vectors^39^ (6) = [0.5257 0.8507 0; 0.5257–0.8507 0; 0 0.5257 0.8507; 0 0.5257–0.8507; 0.8507 0 0.5257; −0.8507 0 0.5257], b = 100, 300, 450 s/mm^2^, repetitions = 30, acquisition time ~29 min. Each site performed imaging with one custom sequence of their preference, including spin echo‐based sequences with up to second‐order motion compensation (M2SE, CODE, MODE, M2B1resist) and STEAM (Figure 2). The relevant acquisition parameters can be found in Table 1 and the supporting information.

**TABLE 1:** Relevant hardware and custom sequence parameters

	Hardware						Pulse sequence (custom)								
Site	B_0_ (T)	Scanner	G_max_ (mT/m)	Max slew rate (T/m/s)	Gradient coil	RF coil	Pulse sequence	TR (ms)	TE (ms)	Diffusion duration,δ_1 …_ δ_n_ (ms)	Time (b – a) (ms)	Slew time, ζ (ms)	Grad Amp, G_1_ … G_n_ (mT/m)	b‐value for non‐DW (b_low_; s/mm^2^)	Crushers during DW scans
A	3	Prisma	80	200	XR	32Ch body + 18Ch spine	M2SE	3000	74	7.8/16	36	1.6	59	5	N
B	3	Prisma	80	200	XR	32Ch body + 18Ch spine	M2 B1resist	3000	97	7.0/14	41	1.0	76	2	Y
C	3	Prisma	80	200	XR	32Ch body + 18Ch spine	M2 B1resist	3000	97	7.0/14	41	1.0	76	2	Y
D	3	Prisma	80	200	XR	32Ch body + 18Ch spine	MODE	3000	75	7.3/17/3.7/13/7.2	36	1.4	65/−65/66/–65/65	0	Y
E	3	Skyra	45	200	XQ	32Ch body + 18Ch spine	CODE	3000	85	11/23/2.3/22/11	42	0.7	−40/40/−40/40/−39	5	N
F	3	Vida	60	200	XT	18Ch body + 18Ch spine	STEAM	2000	35	5.4	1000	0.9	15	42	N
G	3	Skyra	45	200	XQ	18Ch body + 32Ch spine	STEAM	2000	35	4.9	1003	1	16	76	N
H	1.5	Achieva	80/40	100/200	QD	32Ch cardiac (16/16 anterior/posterior)	STEAM	6000	37	4.0	1000	0.21	20	39	N
I	3	Prisma	80	200	XR	18Ch body + 32Ch spine	STEAM	2000	34	4.0	1000	1.5	20	20	N
J	3	Prisma Fit	80	200	XR	32Ch body + 18Ch spine	STEAM	2000	24	2.8	998	0.4	28	16	N

*Note*: The timings and amplitudes of diffusion gradient waveforms (b = 450 s/mm^2^) are stated with reference to the schematic pulse sequence diagrams given in Figure 2. In the case of PGSE and STEAM, time (b – a) corresponds to the diffusion time, Δ.

Abbreviations: CODE, convex optimised diffusion encoding; M2B1resist, M2SE with improved robustness to B_1_ inhomogeneities; M2SE, second‐order motion‐compensated spin echo; MODE, motion‐compensated optimised diffusion encoding; PGSE, pulsed gradient spin echo; STEAM, stimulated echo acquisition mode.

### 2.3 Data analysis

Image reconstruction was performed on the scanners using standard vendor reconstruction software. Data storage and management were performed using the Human Heart Project, an online platform for heart imaging research.^40^, ^41^ Data were analysed centrally using open source Matlab code (https://github.com/vigente/gerardus). The first step was semiautomatic tube segmentation, where tube centres were defined manually, and ROIs generated up to a fixed radius from the tube centres. Tensors were fit directly to the data using linear least squares, using all image repetitions without prior averaging. MD and FA were calculated as follows (Equations 1 and 2, respectively).

(1) $$\mathrm{MD}=\frac{\lambda_{1}+\lambda_{2}+\lambda_{3}}{3}$$

(2) $$\mathrm{FA}=\frac{1}{\sqrt{2}}\sqrt{\frac{{\left(\lambda_{1}-\lambda_{2}\right)}^{2}+{\left(\lambda_{1}-\lambda_{3}\right)}^{2}+{\left(\lambda_{2}-\lambda_{3}\right)}^{2}}{{\lambda_{1}}^{2}+{\lambda_{2}}^{2}+{\lambda_{3}}^{2}}},$$

where λ_1_, λ_2_ and λ_3_ are the principal eigenvalues of the diffusion tensor. To obtain the reference diffusivity of water (D_ref_), the self‐diffusion of water, as measured using an independent non‐MR technique, was extrapolated to 0°C using a second‐order polynomial fit.^42^ To assess the effect of b‐values and rationalise an appropriate choice of b‐values, average values of MD and FA across an ROI in tube 1 using data acquired with different b‐value combinations are reported. Root mean squared differences (RMSDs) between scans 1 and 2 were assessed. MD and FA across ROIs in all tubes were reported using data from all repetitions, and also expressed as a time course reconstructed from subsampled single repetitions. The reference diffusivity for water was defined as *D*_*ref*_ (H_2_O) = 1.113 x 10^−3^ mm^2^/s at 0°C extrapolated from Mills.^42^

To calculate drift, the MD and FA across repetitions were first smoothed with a sliding window of five repetitions. Drift was determined by the difference between final and initial values. The stability of MD and FA measurements across repetitions were expressed in terms of the standard deviation (SD) across repetitions. As the non‐DW data had variable b‐values, image SNR was calculated based on the b = 100 s/mm^2^ images averaged across six DW directions: SNR = mean/SD over repetitions.^43^

Accuracy in MD in each tube and site was expressed as^44^:

(3) $$\text{Accuracy},\partial_{j,k}=\frac{{\bar{\mathrm{MD}}}_{j,k}-{\bar{\mathrm{MD}}}_{\mathrm{ref},j}}{{\bar{\mathrm{MD}}}_{\mathrm{ref},j}}\times 100\%,$$

where $\bar{\mathrm{MD}}$ is averaged across each ROI and both scans. The reference $\bar{\mathrm{MD}}$_*ref*_ is the MD measured by PGSE averaged across each ROI, and all sites and both scans. *j* and *k* are the tube and site indexes.

Precision in MD in each tube and site was expressed in terms of^45^:

(4) $$\text{Coefficient of variation},\;\mathrm{CV}{\left(\mathrm{ROI}\right)}_{j,k}=\frac{{\left[\sigma \left({\mathrm{MD}}_{i}\right)\right]}_{j,k}}{{\left[\mu \left({\mathrm{MD}}_{i}\right)\right]}_{j,k}}\times 100\%,$$

where y *σ* is the SD of MD and *μ* is the mean MD across each ROI in scan 1. *i*, *j* and *k* are the voxel, tube and site indexes, respectively.

Intrasite repeatability was assessed by Bland–Altman plots, with mean differences and 95% limits of agreement reported.

Intersite reproducibility was assessed in terms of:

(5) $$\text{Coefficient of variation},\;{\mathrm{CV}}_{k,l,m}=\frac{\sigma \left({\bar{\mathrm{MD}}}_{k,l,m}\right)}{\mu \left({\bar{\mathrm{MD}}}_{k,l,m}\right)}\times 100\%,$$

where *σ* is the SD of MD and *μ* is the mean MD across each variable separately, $\bar{\mathrm{MD}}$ is averaged across each ROI and both scans. *k* is the site index (1, 2, … 10), *l* is the sequence index (1:PGSE, 2:SE, 3:STEAM), *m* is the scan index (1, 2). We report the interscan, intersite (PGSE, SE, STEAM), intersequence (PGSE vs. SE, PGSE vs. STEAM) and intersite/sequence CV (SE vs. STEAM).

### 2.4 Statistical analysis

Differences in MD with respect to D_ref_ were assessed by one‐sample t‐tests. Shapiro–Wilk tests were performed to test for normality in the accuracy of MD. Wilcoxon rank‐sum tests were performed in each tube to determine the significance of differences between the medians of each sequence with respect to other sequences. The Bland–Altman results were analysed by pairwise comparisons of mean differences between PGSE, SE and STEAM, using two‐sample t‐tests with unequal variances. A significance level of *p* = 0.05 was used. To compare statistical distributions of the reproducibility of MD and FA, bootstrapped histograms were calculated by sampling the differences in MD and FA between scans 1000 times with replacement. Medians and 95% confidence intervals are reported. Histograms with nonoverlapping 95% CIs were deemed to be significantly different from one another.

## 3 RESULTS

### 3.1 Data quality

All sites acquired DTI data at two time points. The custom sequence acquisitions were split evenly into two groups, SE (N = 5) and STEAM (N = 5). While the product sequence acquisitions were standardised apart from small scanner variations in diffusion gradient duration (25.6 ± 2.9 ms) and diffusion time (39.9 ± 3.5 ms; mean ± SD across sites), there was greater variation across the custom sequence acquisitions. The main difference was that of all those sites that acquired STEAM data, only one site (site H) was able to acquire three slices of data as stipulated in the protocol, by slice interleaving and using TR = 6000 ms. To maintain consistent scan time, 15 repetitions were used in the analysis. The other four sites were unable to acquire multislice data in an interleaved fashion because of implementation limitations, and therefore only acquired single‐slice data. As TR = 2000 ms was used, 45 repetitions were acquired to match scan times (sites F, G and I), although one site acquired 30 repetitions (site J). In the custom SE data, 30 repetitions were acquired with TR = 3000 ms, matching the product sequence. There were, however, instances in the custom SE data where not all 30 repetitions were available for all DW directions: 27 (site A, scan 1), 29 (site A, scan 2) and 28 (site E, scan 2) repetitions. Where a given repetition did not include all required DW directions, that repetition was excluded from analysis. In one site (site H), the data were exported at a reconstructed in‐plane resolution of 0.94 x 0.94 mm, and had to be resampled to the nominal 2.5 x 2.5 mm resolution. Variations in parameters such as TR, TE and diffusion gradient timings are given in Table 1.

**FIGURE 3:**
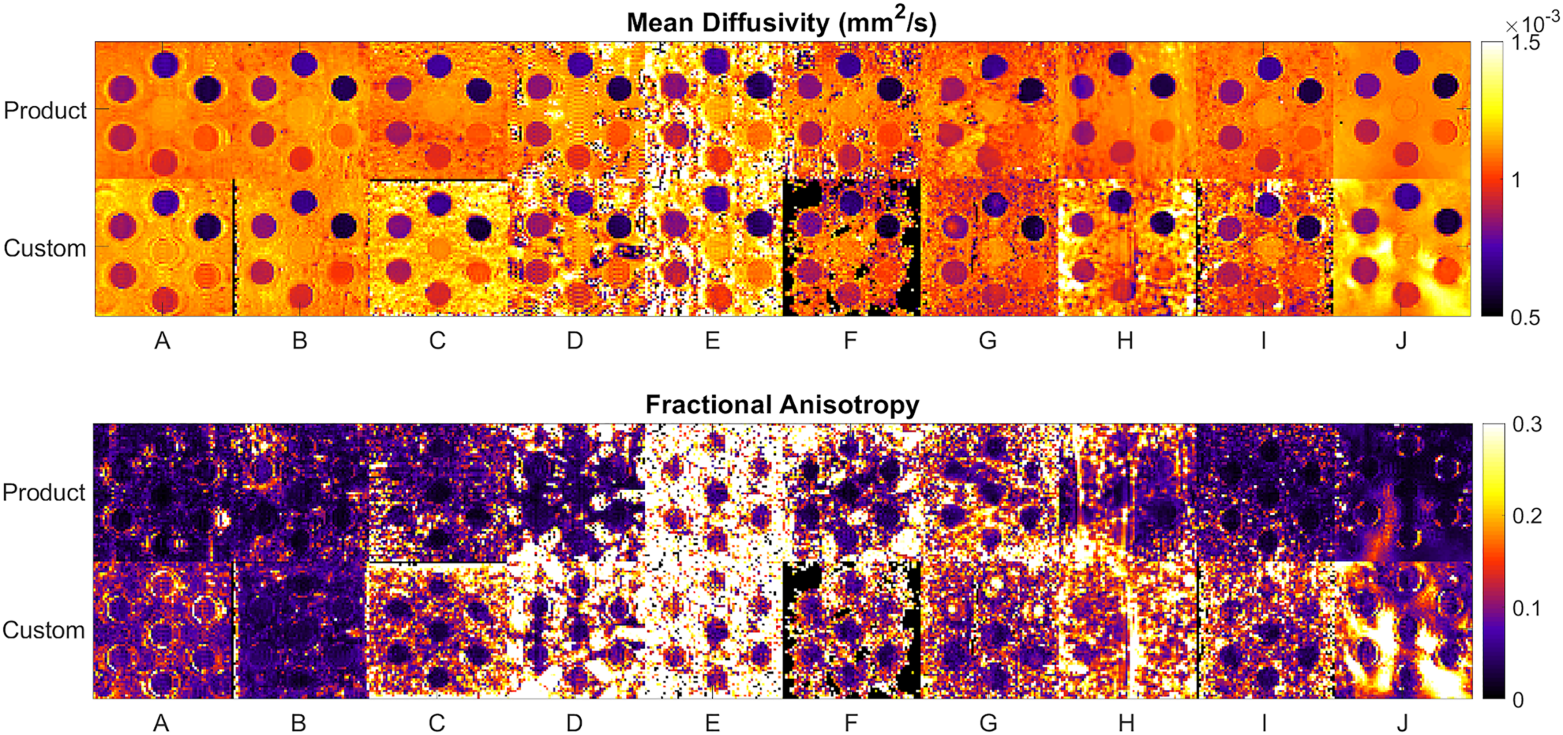
Mosaic of mean diffusivity (MD; top) and fractional anisotropy (FA; bottom) maps. Single‐slice data are shown and were acquired using product (top) and custom sequences (bottom) at scan 1

Figure 3 shows representative MD and FA maps in a single slice acquired at scan 1, using both product and custom sequences. Qualitatively, the maps were of good quality within the tubes. By contrast, the data in the surrounding ice‐water bath were highly variable, and some voxels were masked out by the scanner reconstruction (site F). Gibb's ringing (all sites), geometric distortions (site G) and SENSE unfolding artefacts (site H) were observed.

### 3.2 Mean diffusivity

**FIGURE 4:**
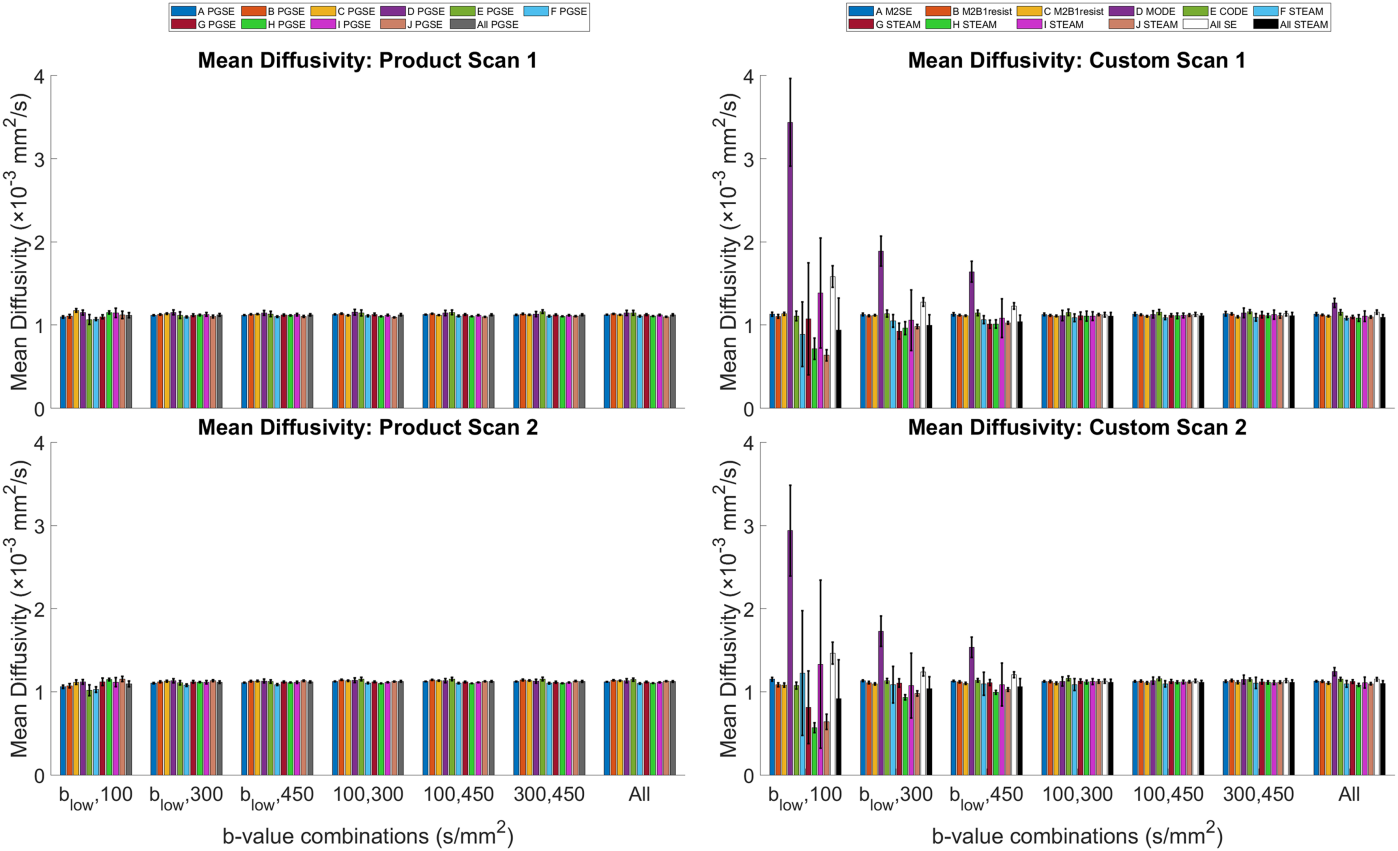
Sensitivity of mean diffusivity (MD) to b‐values used in the diffusion tensor imaging (DTI) reconstruction. Average MD in tube 1 (0% polyvinylpyrrolidone) across sites, two time points, product (left) and custom sequence data (right) and b‐value combinations. Mean ± SD across voxels in the region of interest. Average values for pulsed gradient spin echo (PGSE), spin echo (SE) and stimulated echo acquisition mode (STEAM) are given by grey, white and black bars, respectively. The b‐values of nondiffusion‐weighted data are denoted by b_low_, and ranged from 0 to 76 s/mm^2^ across sites (Table 1). MD and FA reconstructed using pairs of b‐values that include b_low_ are generally further from expected values and less reproducible across scans. The combination of b = (100, 450) s/mm^2^ was considered in subsequent analysis

The dependence of MD on b‐values in the DTI reconstruction is illustrated in Figure 4. The results show that MD calculated from the product sequence data was relatively stable at (1.120 ± 0.022) x 10^−3^ mm^2^/s across all b‐value combinations. By contrast, greater sensitivity to b‐value combinations was observed in MODE and STEAM custom sequences with MD = (1.61 ± 0.73, 1.06 ± 0.13) x 10^−3^ mm^2^/s across b‐value combinations, respectively. The RMSDs of MD between scans 1 and 2 in the custom sequence data were (0.214, 0.078, 0.048, 0.010, 0.005, 0.010, 0.012) x 10^−3^ mm^2^/s across the b‐value combinations b_low_, 100; b_low_, 300; b_low_, 450; 100, 300; 100, 450; 300, 450; and b_low_,100, 300, 450, respectively. The lowest RMSD for MD was found using the combination of b = 100 and 450 s/mm^2^, and this was therefore used for subsequent analysis. As phantom was isotropic, we focus on MD; corresponding FA measurements can be found in the supporting information.

**FIGURE 5:**
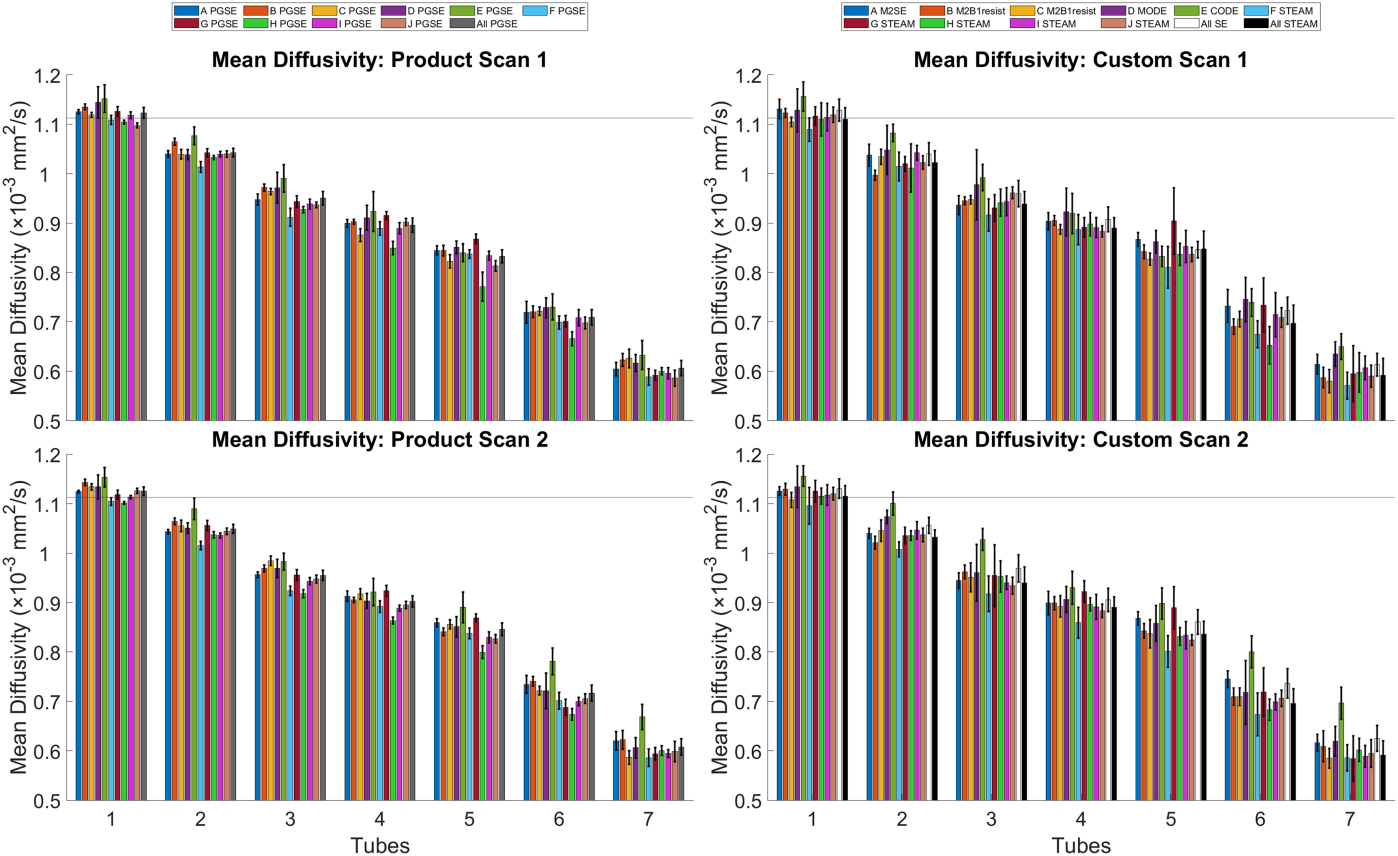
Average mean diffusivity (MD) across regions of interest (ROIs) as a function of polyvinylpyrrolidone (PVP) concentration. Tensors were reconstructed using b = (100, 450) s/mm^2^ data. Tubes 1–7 corresponded to 0%, 2.5%, 5%, 7.5%, 10%, 15% and 20% PVP, respectively. Mean ± SD across voxels in the ROI. The horizontal black line indicates the reference diffusivity (H_2_O at 0°C) = 1.113 x 10^−3^ mm^2^/s

Figure 5 depicts the MD averaged over ROIs within each tube. In tube 1 (0% PVP), the average MDs across all sites and scans 1 and 2 were (1.124, 1.130, 1.113) x 10^−3^ mm^2^/s for PGSE, SE and STEAM, respectively. Relative to the reference diffusivity of H_2_O at 0°C of 1.113 x 10^−3^ mm^2^/s, the average MD of tube 1, as measured by PGSE and SE, was overestimated by 1.0% (*p* = 0.01) and 1.5% (*p* = 0.01), respectively, while STEAM provided a result that was accurate to within 0.04% (*p* = 0.9). The SD across ROIs in tubes 1–7 increased with PVP concentration, particularly in the STEAM data. Summary values of MD and FA grouped by pulse sequence are presented in Table 2, alongside literature values where available.

**TABLE 2:** Mean diffusivity (MD) and fractional anisotropy (FA) across regions of interest in tubes 1–7 by sequence

Tube	[PVP] (%)	MD (x 10^−3^ mm^2^/s)				FA			
		PGSE	SE	STEAM	Literature	PGSE	SE	STEAM	Literature
1	0	1.124 ± 0.017	1.13 ± 0.017^†^	1.113 ± 0.011*	1.115 ± 0.033,^29^ 1.123 ± 0.023,^30^ 1.12 ± 0.01^34^	0.028 ± 0.016	0.052 ± 0.016*	0.059 ± 0.030*	0.037 ± 0.010^29^
2	2.5	1.046 ± 0.018	1.048 ± 0.030	1.027 ± 0.014*	1.05 ± 0.02^34^	0.036 ± 0.023	0.059 ± 0.023*	0.064 ± 0.031*	
3	5	0.953 ± 0.023	0.964 ± 0.028^†^	0.939 ± 0.015	0.98 ± 0.02^34^	0.049 ± 0.034	0.065 ± 0.032	0.069 ± 0.038	
4	7.5	0.899 ± 0.020	0.907 ± 0.014^†^	0.890 ± 0.016		0.042 ± 0.024	0.060 ± 0.021*	0.066 ± 0.023*	
5	10	0.839 ± 0.026	0.854 ± 0.022	0.842 ± 0.032	0.850 ± 0.024,^30^ 0.85 ± 0.01^34^	0.049 ± 0.036	0.062 ± 0.034	0.090 ± 0.047*	
6	15	0.713 ± 0.025	0.729 ± 0.031^†^	0.697 ± 0.015	0.71 ± 0.01^34^	0.052 ± 0.032	0.078 ± 0.042	0.113 ± 0.066*	
7	20	0.607 ± 0.021	0.619 ± 0.035^†^	0.592 ± 0.010*	0.607 ± 0.019,^30^ 0.59 ± 0.02^34^	0.048 ± 0.028	0.078 ± 0.032*	0.123 ± 0.067*	

*Note*: MD and FA given as mean ± SD across sites and time points. Values from the literature using PGSE at a single time point are given for matching PVP concentrations and temperature. Significant differences at *p* < 0.05 (i) in SE and STEAM with respect to PGSE are denoted by *, and (ii) in SE with respect to STEAM by ^†^.

Abbreviations: PGSE, pulsed gradient spin echo; PVP, polyvinylpyrrolidone; SE, spin echo; STEAM, stimulated echo acquisition mode.

### 3.3 Accuracy

**FIGURE 6:**
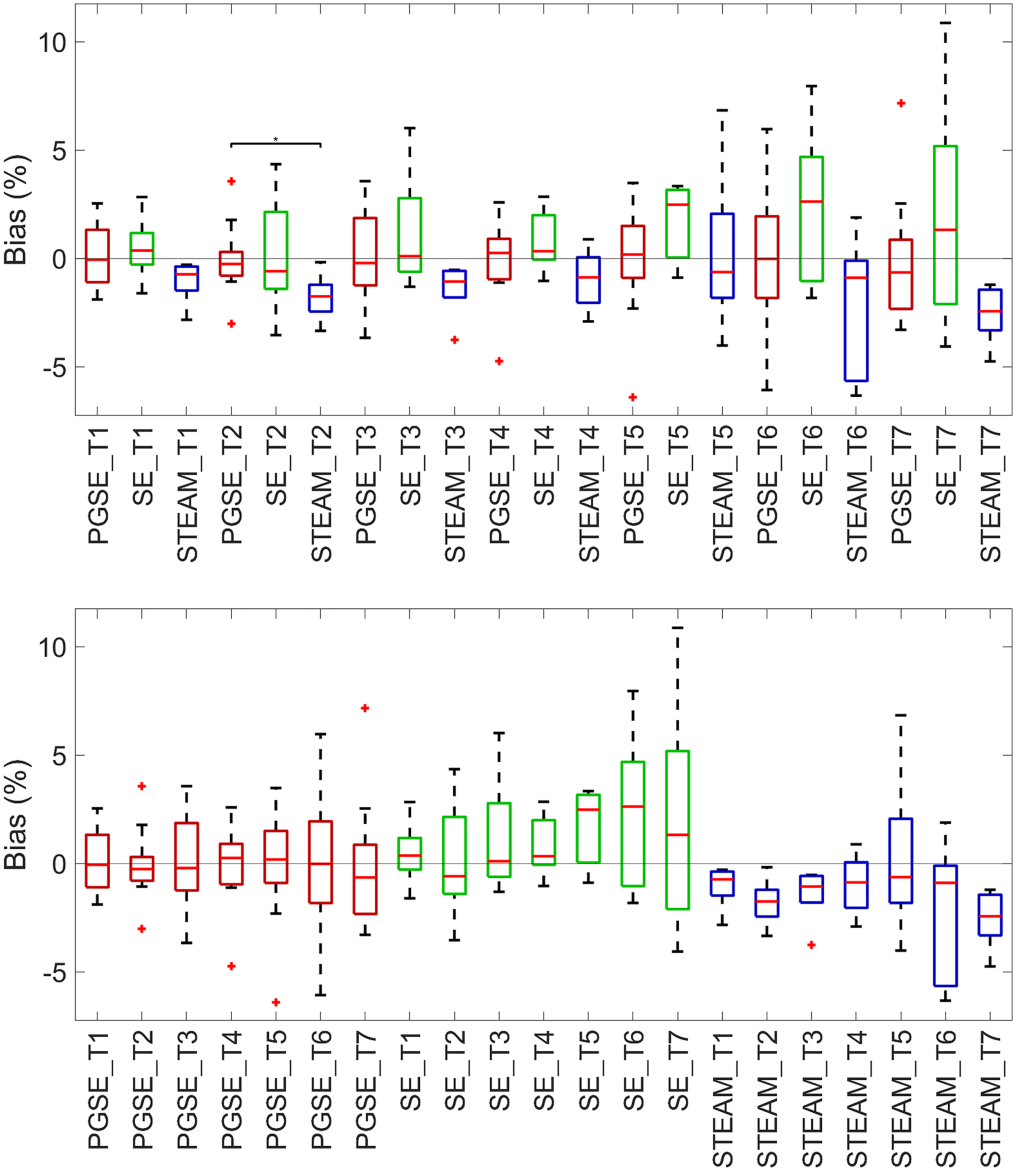
Accuracy in mean diffusivity (MD) measurements. Accuracy of MD relative to the reference MD was reported across pulse sequences and tubes (T). The reference MD in each tube was determined by the mean MD across sites and scans. Data from scans 1 and 2 were first averaged. Wilcoxon rank‐sum tests were performed between pairs of sequences (pulsed gradient spin echo [PGSE] vs. spin echo [SE]; SE vs. stimulated echo acquisition mode [STEAM]; PGSE vs. STEAM) for each separate tube, and between pairs of tubes for each separate sequence; *p < 0.05). Differences in median accuracy between tubes within the same sequence were not significant at *p* < 0.05. Data were sorted by sequences‐tubes (top) and tubes‐sequences (bottom) for clarity

The accuracy of MD relative to the average MD measured across sites is presented in Figure 6. The null hypothesis that samples were normally distributed was rejected in three cases (STEAM tube 1, SE tube 3 and PGSE tube 7). Median accuracies between sequences were not significantly different, except for PGSE tube 2 versus STEAM tube 2 (*p* < 0.05). Within pulse sequences and between tubes, there were no significant differences in median accuracies.

### 3.4 Precision

**FIGURE 7:**
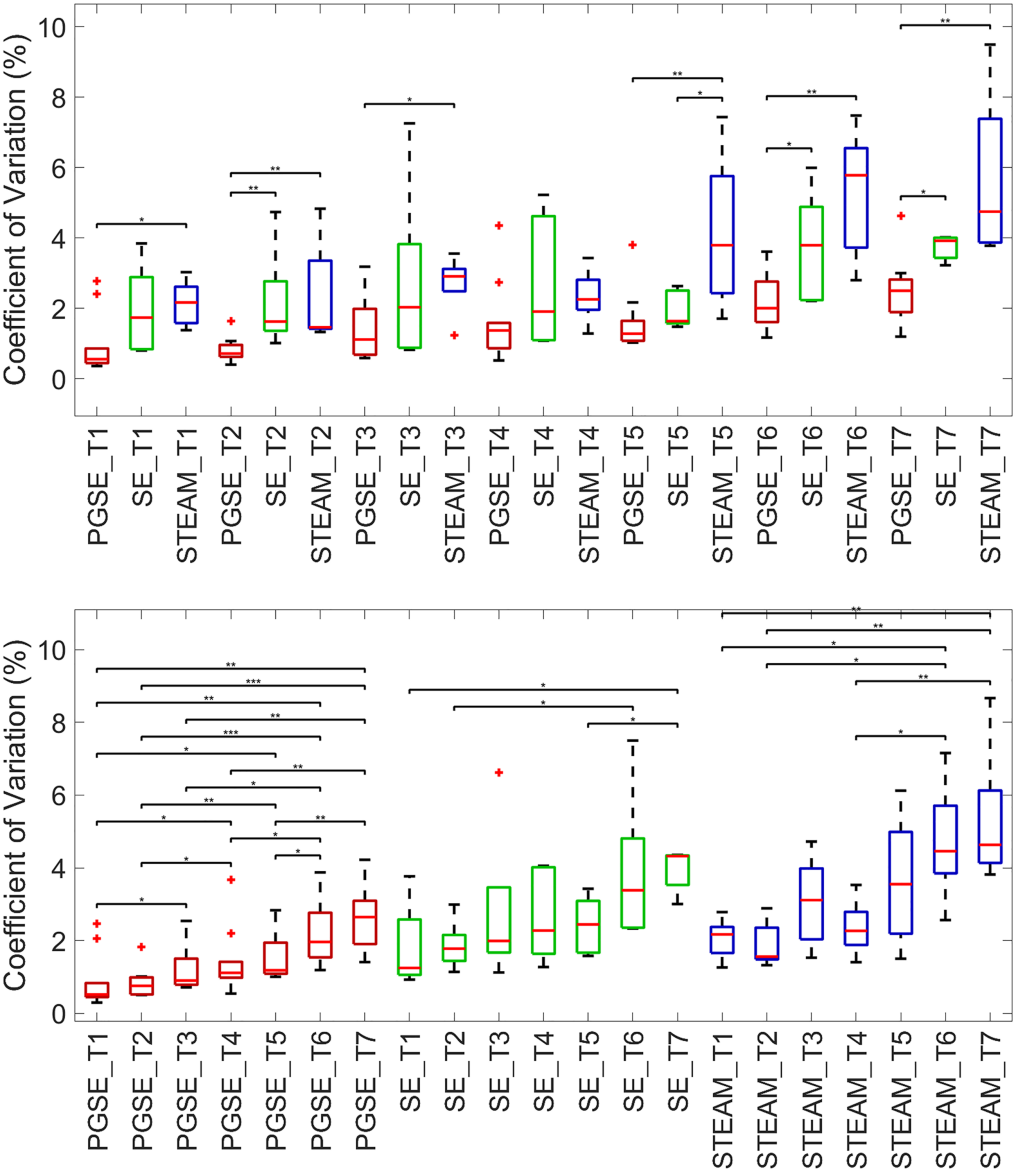
Precision in mean diffusivity (MD) measurements. Precision of MD was reported across pulse sequences and tubes (T). Wilcoxon rank‐sum tests were performed between pairs of sequences and tubes; *p < 0.05, **p < 0.01, ***p < 0.001. In several tubes, precision was poorer in spin echo (SE) and stimulated echo acquisition mode (STEAM) compared with pulsed gradient spin echo (PGSE). There was a clear dependence of precision on tube number. Data were sorted by sequences‐tubes (top) and tubes‐sequences (bottom) for clarity

The precision of MD across pairs of sequences and tubes show that SE has significantly higher median CV than PGSE in three tubes, whereas STEAM has significantly higher median CV than PGSE in six tubes (Figure 7). There were clear differences as well when comparing medians between pairs of tubes, with precision worsening with increasing tube number (i.e. PVP concentration).

### 3.5 Intrascan stability

**FIGURE 8:**
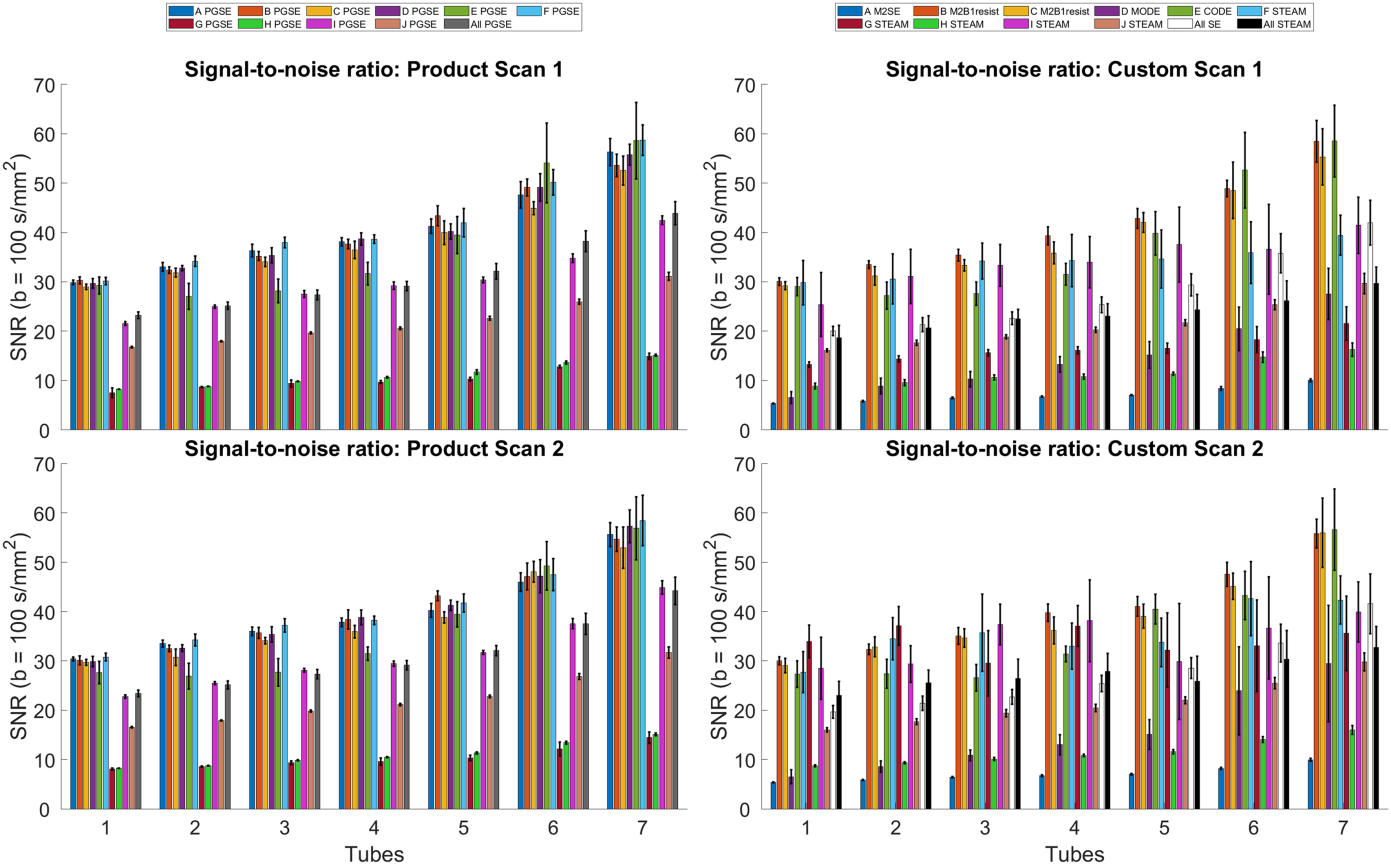
Signal‐to‐noise ratio (SNR) of b = 100 s/mm^2^ images. SNR was highest in the pulsed gradient spin echo (PGSE) data: 23.3 and 44.1 in tubes 1 and 7, respectively. Relative to PGSE, SNRs (spin echo [SE]) in tubes 1 and 7 were 85.1% and 94.8%, respectively, while SNRs (stimulated echo acquisition mode [STEAM]) were 89.4% and 70.8%, respectively. Mean ± SD across voxels in the region of interest

Figure 8 shows the SNR of the b = 100 s/mm^2^ data in a single repetition. In tube 1, the average SNRs across scans 1 and 2 were 23.3, 19.9 and 20.8 in PGSE, SE and STEAM, respectively, whereas in tube 7, the corresponding average SNRs were 44.1, 41.8 and 31.2. The stability of measurements across repetitions is reported in the supporting information.

### 3.6 Intrasite repeatability

**FIGURE 9:**
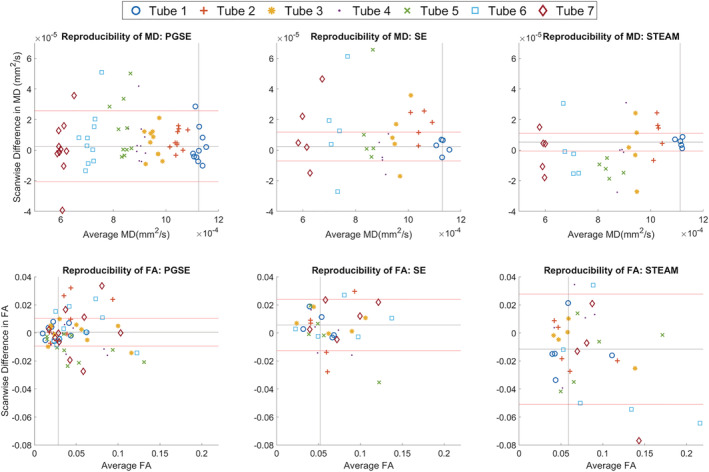
Bland–Altman plots of mean diffusivity (MD) and fractional anisotropy (FA) across two scans (scan 2 – scan 1). Data acquired using pulsed gradient spin echo (PGSE; left), spin echo (SE; middle) and stimulated echo acquisition mode (STEAM; right) sequences in tubes 1 to 7 (0%–20% polyvinylpyrrolidone [PVP]) are colour‐coded by increasing PVP concentration. The black and red horizontal lines indicate the mean and ±1.96 SD values for tube 1, respectively. The black vertical line indicates the average values for tube 1 across sites

**FIGURE 10:**
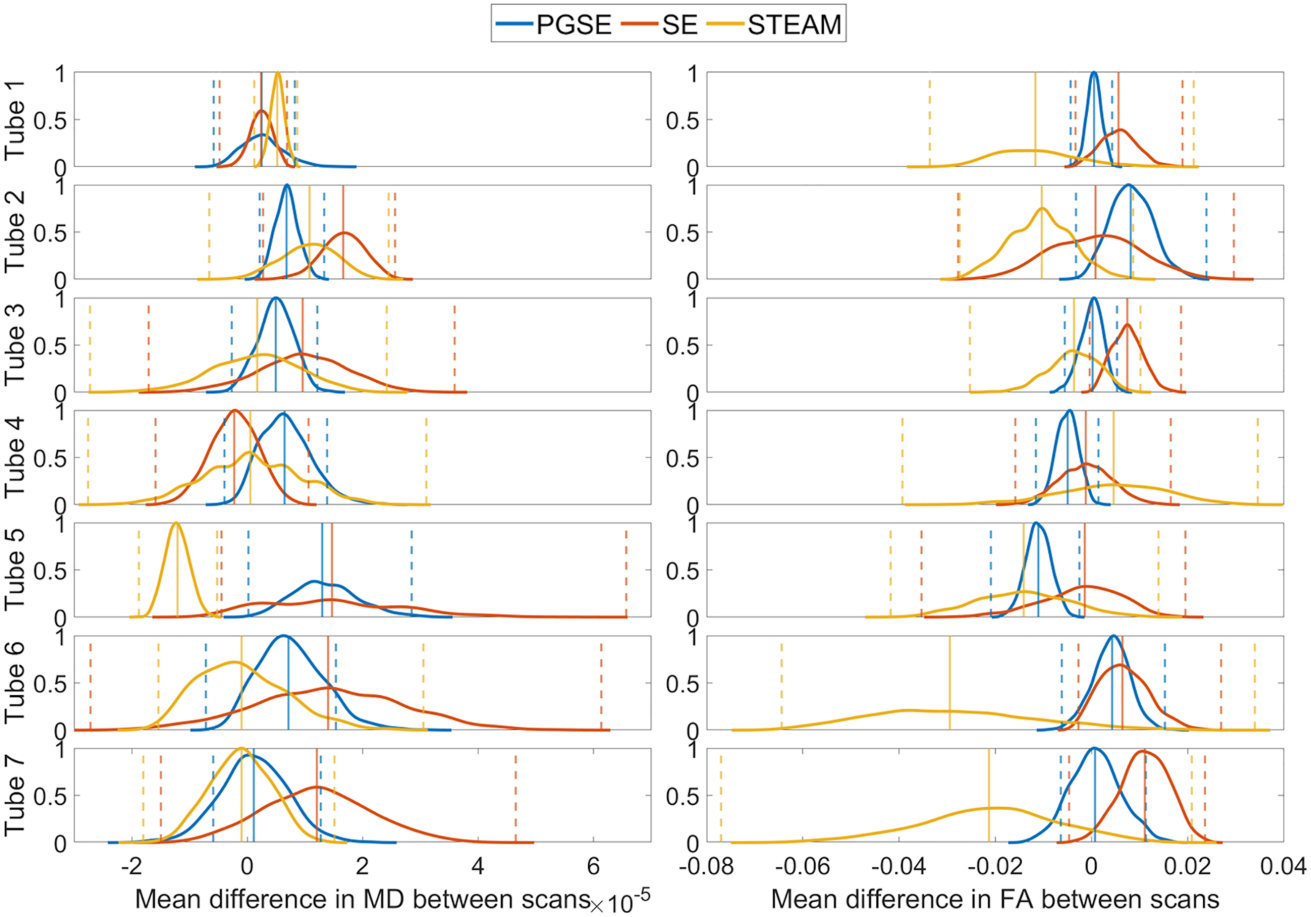
Bootstrapped normalised histograms of mean differences in mean diffusivity (MD) and fractional anisotropy (FA) between scans. Median and 95% confidence intervals (CIs) are given by vertical solid and dashed lines. The MD difference in stimulated echo acquisition mode (STEAM) was significantly lower than that of pulsed gradient spin echo (PGSE) and spin echo (SE) in tube 5. All other differences in reproducibility between sequences were not significant

Bland–Altman plots of MD and FA across two scans are presented in Figure 9. In tube 1, the mean differences of MD in PGSE, SE and STEAM were (0.3 ± 2.3, 0.24 ± 0.95, 0.52 ± 0.58) x 10^−5^ mm^2^/s, respectively (mean ± 1.96 SD). The corresponding mean differences in FA were 0.0006 ± 0.0099, 0.006 ± 0.018 and −0.012 ± 0.039. The differences between pulse sequences, as observed in the mean differences of MD and FA, were not significant at *p* = 0.05. Linear regression of difference by average values of MD and FA showed low proportional bias, with R^2^ of 0.16 or less in all cases. Figure 10 summarises the bootstrapped scan‐wise mean differences in MD and FA, grouped by pulse sequence. On average and across tubes, the greatest median scan‐wise difference in MD of 0.96 x 10^−5^ mm^2^/s was seen in the SE data, whereas STEAM exhibited the greatest median scan‐wise difference in FA of −0.012. Table S1 compares the mean difference averaged across all tubes across sites. The lowest absolute mean differences in MD and FA in the custom sequence data were seen in sites A and D, respectively.

Overall, STEAM yielded more accurate MD than SE at the isocentre (*p* = 0.02), while MD and FA across repetitions were more stable in SE compared with STEAM (*p* < 0.001). Although SE tended to higher reproducibility in MD and FA than STEAM, these were not significant at *p* = 0.05.

### 3.7 Intersite reproducibility

**FIGURE 11:**
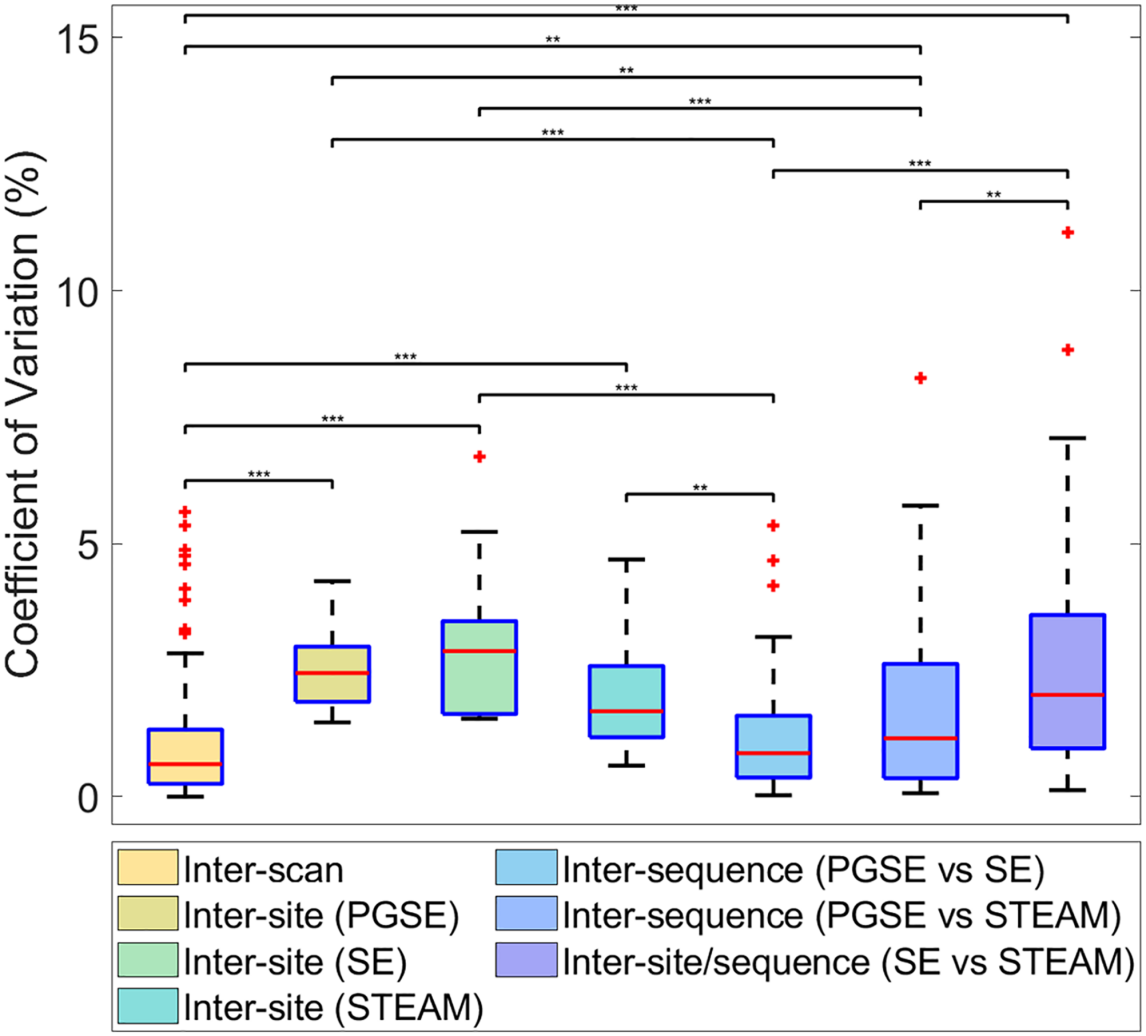
Reproducibility of mean diffusivity (MD) across scans, sites and sequences. Coefficients of variation in MD across scans, sites and sequences. Wilcoxon rank‐sum tests were performed pairwise between groups; **p < 0.01, ***p < 0.001. PGSE, pulsed gradient spin echo; SE, spin echo; STEAM, stimulated echo acquisition mode

The average CV_MD_ was evaluated across scans, sites (PGSE, SE and STEAM separately) and sequence pairs (PGSE vs. SE, PGSE vs. STEAM and SE vs. STEAM). The results were: CV_MD(scans)_ = 1.0%, CV_MD(sites, PGSE)_ = 2.6%, CV_MD(sites, SE)_ = 3.1%, CV_MD(sites, STEAM)_ = 2.1%, CV_MD(sequences, PGSE vs SE)_ = 1.2%, CV_MD(sequences, PGSE vs. STEAM)_ = 1.6% and CV_MD(sequences, SE vs. STEAM)_ = 2.6% (Figure 11). Within the intersite grouping, there were no significant differences in median CV between different sequences. Within the intersequence grouping, median CV in SE versus STEAM was significantly higher than both PGSE versus SE and PGSE versus STEAM.

## 4 DISCUSSION

In this study, we compared several state‐of‐the‐art methods suitable for in vivo cardiac DTI alongside conventional PGSE data. The results at the isocentre (i.e. tube 1) showed that, averaged across sites, PGSE, SE and STEAM yielded accurate MD, agreeing with the reference diffusivity of water at 0°C to within 1.5% or less. Moreover, the MDs in the PGSE, SE and STEAM data were within ±1% of averaged literature values,^33^, ^34^, ^37^ suggesting good conformance with prescribed experimental conditions. The diffusivity of PVP depends on the concentration, temperature and length of polymer chains, and an independently measured ground truth is unavailable. We therefore used the MD acquired using PGSE averaged across sites and scans as a reference for assessing accuracy in PVP with different concentrations: these values were in good agreement with the literature (Table 2).

The expected value of FA in an isotropic phantom is zero. However, factors including noise, imperfect gradient calibration, eddy currents, and convection and vibration within the phantom could in principle contribute to nonzero FA. Both SE and STEAM had lower SNRs than PGSE, which could have resulted in the higher FA in the custom sequences. We chose not to report CV_FA_ because the expected FA in isotropic media is zero, which leads to unstable CV_FA_ that is biased by the measured FA. For instance, poor gradient calibration would lead to a higher FA, and therefore artificially lower CV_FA_. Vibration and convection were not deemed to be major issues due to the good accuracy of MD.

CV (ROI) in MD and FA across sites was lowest in PGSE and highest in STEAM. In an isotropic liquid phantom, the underlying substrate is expected to be homogeneous. Reasons that could increase CV (ROI) include noise and image artefacts. From Figure 3, Gibb's ringing can be seen in all datasets due to the hard edges of the phantom. Additional artefacts are visible in the STEAM data (sites G and H), potentially increasing the CV (ROI). Furthermore, as the tube number and PVP concentration increased, we observed that the mean FA and CV (ROI) in MD and FA were increasingly elevated, particularly in STEAM. This may be associated with (i) effects of concomitant fields away from the isocentre (site E), (ii) localised image artefacts in tubes 5, 6 and 7 possibly caused by vibration (site G), and (iii) the increased effect of Gibb's ringing in the tubes with higher PVP concentrations due to the increased differential in DW image intensities relative to the surrounding ice‐water bath.

In cardiac DTI, DW data with finite, low b‐values are often used instead of non‐DW data to suppress the effects of microvascular perfusion.^23^ There was no perfusion in the phantom, and therefore MD and FA would be expected to be relatively insensitive to the b‐value combinations used, insofar as perfusion is concerned. This was generally the case for PGSE data. Greater deviations were seen when custom DTI data were reconstructed from b‐value pairs that included non‐DW data. See b_low_ in Table 1 for corresponding b‐values. Deviations from the expected values of MD and FA were particularly striking in MODE and STEAM data. With MODE, high MD was seen whenever the b_low_ data were used. This was due to a shading artefact seen in the DW data, but not in the non‐DW data, therefore resulting in a positive bias in MD when the non‐DW data were included in the reconstruction. The shading artefact was potentially due to residual concomitant gradient field effects, and this was reflected in higher regional heterogeneity compared with other SE data. In STEAM, the value of b_low_ can be substantial due to the effect of crushers and long diffusion times. The diffusion contrast between b_low_ and b_high_ = 100 s/mm^2^ can therefore be insufficient for reliable estimation of MD and FA. This is a general result of the increased effects of noise as the range of b‐values used for reconstruction are reduced, for instance, resulting in a positive bias in FA. As b_high_ increased to 450 s/mm^2^, the errors were reduced. Where combinations of b = (100, 300), (100, 450) and (300, 450) s/mm^2^ were used, the MD and FA were qualitatively indistinguishable. However, we found that the combination of b = (100, 450) s/mm^2^ yielded the best reproducibility in MD between scans 1 and 2, and this was consistent with typical b‐values used in the literature.^19^, ^22^

The SNRs in the b = 100 s/mm^2^ images at the isocentre were comparable between SE and STEAM. The SNR generally increased with PVP concentration as the mean signal intensity was less attenuated by diffusion‐weighting. Away from the isocentre in tube 7, the image SNRs in PGSE and SE were 41% and 34% higher than that in STEAM, which may have arisen from image artefacts as described (sites G and H). Furthermore, in four instances of STEAM, only a single slice was acquired due to limitations in implementation. In these cases, reduced FOV was used, which involved excitation slice‐selection gradients that were orthogonal to subsequent slice‐selection gradients. As a result, acquisition of multiple slices while maintaining constant acquisition time would necessitate a reduction in the number of repetitions proportional to the number of slices (site H). By implementing a slab‐tilted reduced FOV imaging method that is compatible with multislice imaging, this enabled site H to extend the TR to 6 s, rather than the 2 s used by the other STEAM sites, which recovers some of the SNR efficiency lost by using the STEAM technique. This motivates a shift from standard slab‐perpendicular methods towards better methods of reduced FOV imaging, such as slab‐tilted methods. We note that image SNR does not fully reflect the stability of measurements, as it does not take into account higher b‐value data and associated potential issues. For instance, among the STEAM acquisitions, lower SNR was found alongside lower CV in MD and FA (site H).

The mean differences in MD in tube 1 across scans were relatively small at 0.22%, 0.21% and 0.47% in PGSE, SE and STEAM, respectively, expressed as a percentage of the respective average MD. The 95% limits of agreement were higher at ±0.84% in SE compared with ±0.52% in the STEAM data. For FA, both the absolute mean difference and 95% limits of agreement were lower in SE (0.006 ± 0.018) compared with STEAM (−0.012 ± 0.039). The MD data suggest that there was good reproducibility across the different classes of sequences. That the reproducibility with respect to FA was poorer could be due to the intrinsically low FA and lack of underlying microstructure.

Intrasite repeatability was superior to intersite and intersequence reproducibility, as measured in terms of CV_MD_. This is consistent with the literature. Intersite reproducibility ranging from 2.1% (STEAM) to 3.1% (SE) was similar to other studies using PGSE alone,^33^, ^34^ where values ranged from 2.1% to 3%. We also observed that intersite reproducibility was marginally poorer than intersequence reproducibility. The CV in all cases was low compared with CV in vivo,^29^ as the phantoms were static, isotropic and consistently fabricated. We would anticipate greater variation between sites and sequences, with added phantom complexity simulating anisotropy and/or motion.

While we consider the various SE sequences collectively, it is worth noting that each of the four SE sequences used have their specific characteristics. Despite having the longest TE, the M2B1resist sequence had the lowest FA, regional heterogeneity in MD and FA, and SD_MD_ and SD_FA_ across repetitions, and highest SNR among custom sequences. Owing to the symmetric design of the diffusion gradients, the sequence, along with M2SE, is inherently more robust to concomitant fields. Furthermore, as the zeroth and first gradient moments are nulled prior to the refocusing pulse, its insensitivity to motion with constant velocity is less dependent on the refocusing pulse being close to 180° compared with other custom SE sequences.^46^ However, the additional gradient lobes relative to M2SE, and the need for crushers due to the nulling of zeroth gradient moments before the refocusing pulse, extend the minimum TE. M2SE and MODE yielded the best interscan reproducibility in MD and FA, respectively. While shorter TE are feasible with CODE and MODE compared with M2SE and M2B1resist, the former two sequences are sensitive to spatially varying concomitant fields due to the asymmetry of diffusion gradient waveforms about the refocusing pulse, and corrections are employed to mitigate image artefacts arising from concomitant fields. As the custom SE group data include data acquired with four different SE sequences, the within‐group variation would be expected to decrease were a single sequence used.

The current work provides a promising basis for future clinical studies. While other quantitative CMR techniques have arguably been hampered by methods that differ even in phantoms and are very sensitive to readout parameters,^47^ here we demonstrate that a wide range of sequences from different centres have excellent agreement in a simple phantom. Clinically, the availability of multiple sequences for cardiac DTI offers sites the flexibility to choose the sequence to suit the pathology of interest and hardware configuration available. SE, for instance, yields higher SNR efficiency but requires higher performance gradient systems. STEAM, on the other hand, is able to acquire data over a wider range of cardiac phases, but requires breath‐holding, which may be difficult in some patient cohorts. While the comparison of cardiac DTI sequences remains an active area of research, there may be a clinical case for both classes of sequences.

There are several limitations of using a static isotropic phantom for quantifying the performance of cardiac DTI sequences. First, the phantom lacks motion. This prevents the assessment of the quality of motion compensation in the custom sequences, where inadequate motion compensation is a primary reason for rejected images, elevated MD and failed scans. Incorrect triggering could be difficult to identify from the static phantom images. Second, the phantom lacks anisotropic microstructure, leading to an expected FA of zero. This results in non‐Gaussian distribution of errors in FA, and enhancement in the errors when expressed as a percentage. Third, the phantom substrate is liquid and therefore could be prone to scanner vibration and thermal convection, which in turn could increase MD, although this was not observed to be a major issue. This effect may be more pronounced in STEAM, where diffusion times are an order of magnitude longer. Fourth, the T_1_ and T_2_ relaxation times of the liquid substrate are different compared with that found in the heart,^37^, ^48^ with T_1,H2O@0°C_ = 1525 ms, T_2,H2O@0°C_ = 1472 ms, T_1,20%PVP@0°C_ = 753 ms, T_2,20%PVP@0°C_ = 623 ms, T_1,Heart in vivo_ = 1184 ms and T_2,Heart in vivo_ = 52 ms, as measured at 3 T. This translates to higher SNR in the phantom relative to heart, and sequences with longer TE such as custom SE, would suffer greater SNR penalties in vivo. Adjusting for T_1_ and T_2_ in the in vivo setting relative to iced water, the signal in SE would be expected to decrease by 73% to 82%, while the signal in the STEAM data would decrease by 29% to 49% based on TR and TE in the custom sequences. This would lead to a theoretical 2.7x increase in signal in STEAM relative to SE in tube 1. In 20% PVP, the relative theoretical improvement in signal in STEAM over SE would be 2.4x.

Despite its shortcomings, the use of a static, isotropic phantom is an important first step for assessing custom sequence performance in a multicentre study, as it allows a baseline assessment of parameters in the absence of additional variables associated with tissue microstructure and motion. The phantom was robust and consistent, having been manufactured centrally, and stable over time, permitting repeated scanning with negligible change in substrate. The use of the ice‐water bath permitted accurate temperature control, which is important, as every 1°C increase in temperature at 0°C leads to an ~5% increase in diffusivity. More elaborate systems for monitoring and maintaining accurate temperature are feasible, but would be costly to implement. In this ice‐water phantom, the maximum MD achievable was ~1.1 x 10^−3^ mm^2^/s in water. This was in the middle of the range of MDs measured in the heart in vivo with SE and STEAM (0.87 x 10^−3^ to 1.72 x 10^−3^ mm^2^/s), and attributable to the low temperature. To simulate higher MD, a higher temperature would be needed. At 35°C, water would have too high an MD (3.0 x 10^−3^ mm^2^/s). Instead, PVP solutions with concentrations (20% to 40%) would cover an appropriate MD range (0.9 x 10^−3^ to 1.8 x 10^−3^ mm^2^/s), based on a previous study.^49^ Crucially, there exist gold standard, independent reference measurements of MD in water over a range of temperatures, and corroborating values of MD in temperature‐controlled PVP phantoms in the literature.

## 5 CONCLUSIONS

In summary, we have validated the accuracy, precision, repeatability and reproducibility of state‐of‐the‐art custom sequences for in vivo cardiac DTI. This study benchmarks the performance of custom SE and STEAM against product PGSE sequences, and identifies baseline variation across sites, scanners and sequences. Some areas of inconsistency in the measurements have been highlighted, which warrant further methodological refinement. Future work includes the development of more sophisticated phantoms with more physiological relaxation times, diffusion anisotropy and motion characteristics. This would add further insight into the robustness and behaviour of cardiac DTI sequences, and the availability of appropriate phantoms will be key to facilitating quality assurance protocols. Quantitative validation of pulse sequences in phantoms represents an important step towards rationalising pulse sequences, and will contribute to protocol harmonisation and the establishment of cardiac DTI in the clinical setting.

## CONFLICT OF INTEREST

Royal Brompton Hospital has research collaboration agreements with Siemens AG Medical Solutions.

## AUTHOR CONTRIBUTIONS

Study conception (AS, CTS, DBE, IT, JES, MV), study coordination and drafting of manuscript (IT); phantom design and build (IT, JB); data acquisition (AS, BR, CN, CT, CTS, DL, EMT, IT, JCF, KM, PK, MV); data analysis (IT, WR); study design and planning, and manuscript review (all). All the authors have approved the submitted version and any substantially modified version that involves each author's contribution to the study, and have agreed both to be personally accountable for their own contributions and to ensure that questions related to the accuracy or integrity of any part of the work are appropriately investigated, resolved, and the resolution documented in the literature.

## Supporting information


**Figure S1.** Intra‐scan stability of MD and FA across repetitions.
Time course of MD and FA in Tube 1 (0% PVP) reconstructed from single repetitions of b = (100,450) s/mm^2^ with 6 DW directions. MD measurements were generally stable over repetitions with drift < 0.5% as averaged across PGSE, SE and STEAM data. The drift in FA across repetitions was larger, averaging +2.4%, +1.1% and +1.2% across PGSE, SE and STEAM data. Considerable FA drift was seen in specific sites and in the product sequence data, where|drift (FA)| > 10% in 4 sites using the product sequence, and none using the custom sequences.

**Figure S2.** Intra‐scan stability of MD and FA.
Values expressed as standard deviation across repetitions. SD_MD_ and SD_FA_ were highest in the STEAM data, and this effect was more pronounced at higher PVP concentrations.

**Figure S3.** Sensitivity of FA to b‐values used in the DTI reconstruction.
Average FA in Tube 1 (0% PVP) across sites, 2 timepoints, sequences and b‐value combinations are shown. The two timepoints are denoted by the numerical suffix in the figure legends; product (left) and custom sequence data (right) are shown. Average values for PGSE, SE and STEAM are given by grey, white and black bars respectively. The b‐values of non‐DW data are denoted by b_low_, and ranged from 0 to 76 s/mm^2^ across sites (Table 1).

**Figure S4.** Average FA across ROIs as a function of PVP concentration.
Tensors were reconstructed using b = (100,450) s/mm^2^ data. Tubes 1–7 corresponded to (0, 2.5, 5, 7.5, 10, 15, 20) % PVP respectively. The expected value of FA in isotropic media is zero. An increasing trend in FA is observed with increasing PVP concentration.

**Table S1.** Mean difference between Scans 1 and 2, averaged across all tubes.

**Data S1.** Supporting Information

## Data Availability

The datasets generated from the current study are available on the Human Heart Project repository https://humanheart-project.creatis.insa-lyon.fr/MultiCentreEvaluationCDTI.html.
