# Supplementary figures and images for: Validation of cardiac diffusion tensor imaging sequences: A multicentre test–retest phantom study
Source: NMR Biomed. 2022 Feb 8;35(6):e4685. doi: 10.1002/nbm.4685 (PMC9285553; doi:10.1002/nbm.4685)

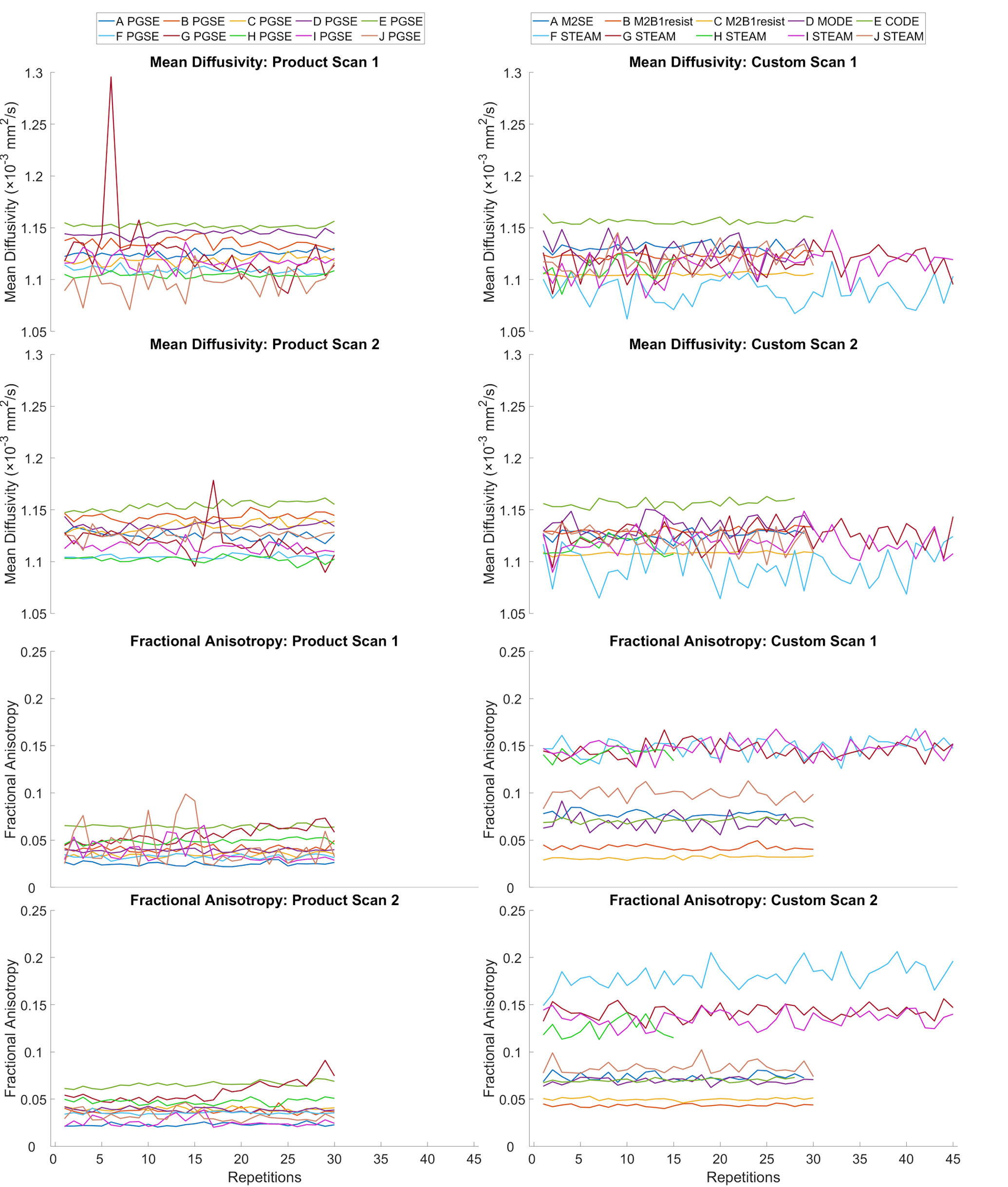

Supplement: Supplementary file 1 — Figure S1. Intra‐scan stability of MD and FA across repetitions. Time course of MD and FA in Tube 1 (0% PVP) reconstructed from single repetitions of b = (100,450) s/mm2 with 6 DW directions. MD measurements were generally stable over repetitions with drift < 0.5% as averaged across PGSE, SE and STEAM data. The drift in FA across repetitions was larger, averaging +2.4%, +1.1% and +1.2% across PGSE, SE and STEAM data. Considerable FA drift was seen in specific sites and in the product sequence data, where|drift (FA)| > 10% in 4 sites using the product sequence, and none using the custom sequences. [file NBM-35-0-s003.tif]

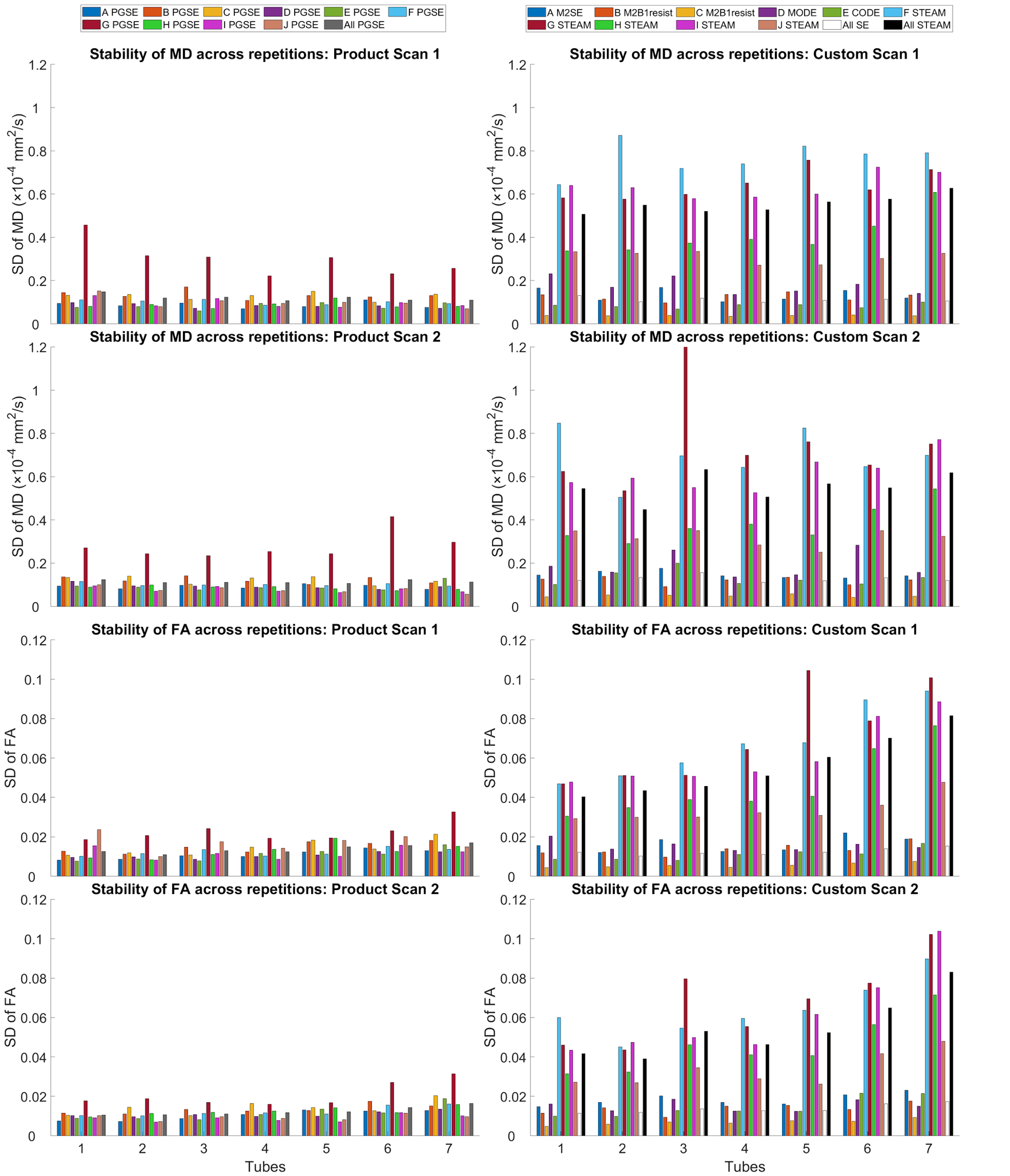

Supplement: Supplementary file 2 — Figure S2. Intra‐scan stability of MD and FA. Values expressed as standard deviation across repetitions. SDMD and SDFA were highest in the STEAM data, and this effect was more pronounced at higher PVP concentrations. [file NBM-35-0-s005.tif]

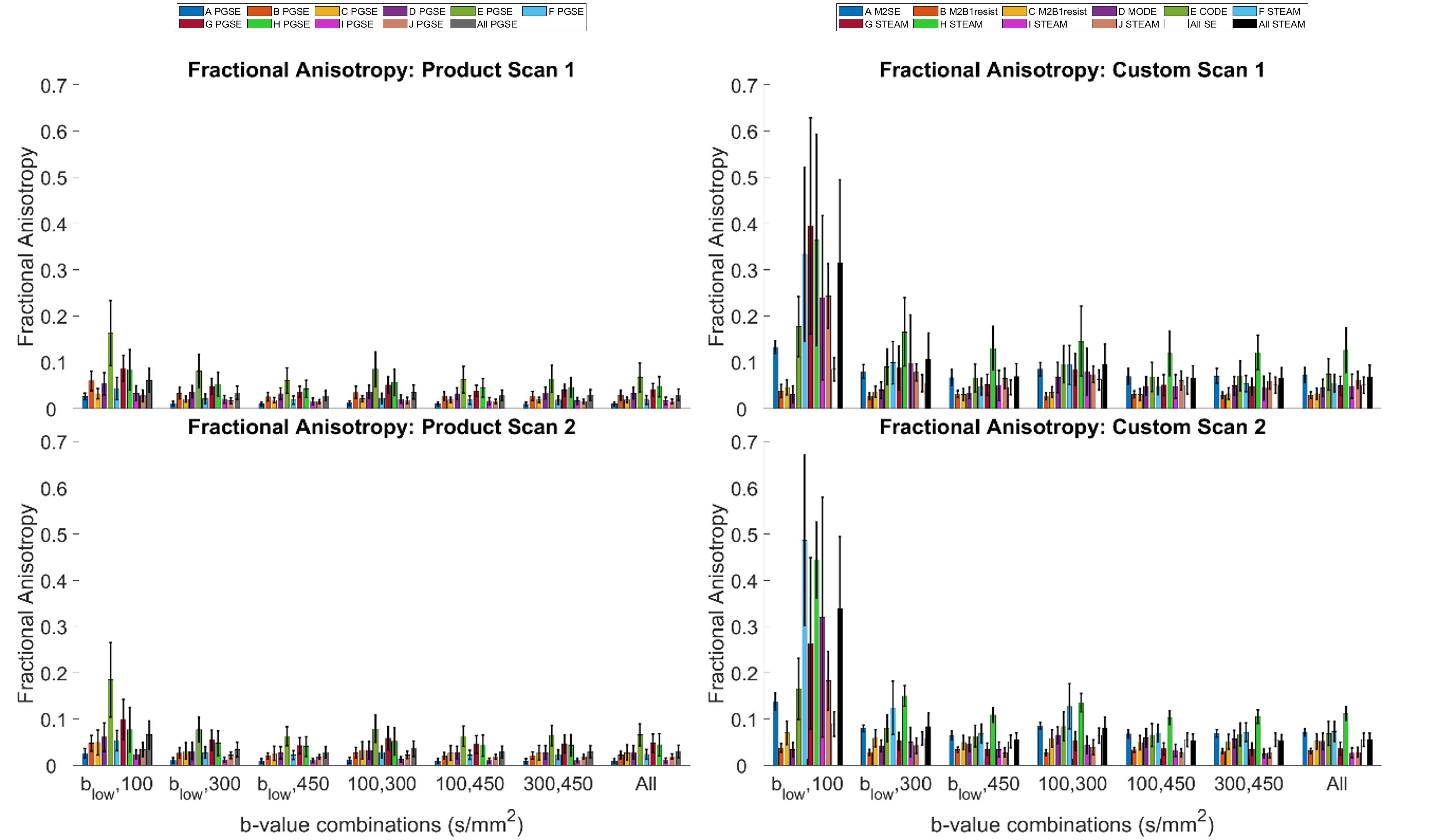

Supplement: Supplementary file 3 — Figure S3. Sensitivity of FA to b‐values used in the DTI reconstruction. Average FA in Tube 1 (0% PVP) across sites, 2 timepoints, sequences and b‐value combinations are shown. The two timepoints are denoted by the numerical suffix in the figure legends; product (left) and custom sequence data (right) are shown. Average values for PGSE, SE and STEAM are given by grey, white and black bars respectively. The b‐values of non‐DW data are denoted by blow, and ranged from 0 to 76 s/mm2 across sites (Table 1). [file NBM-35-0-s001.tif]

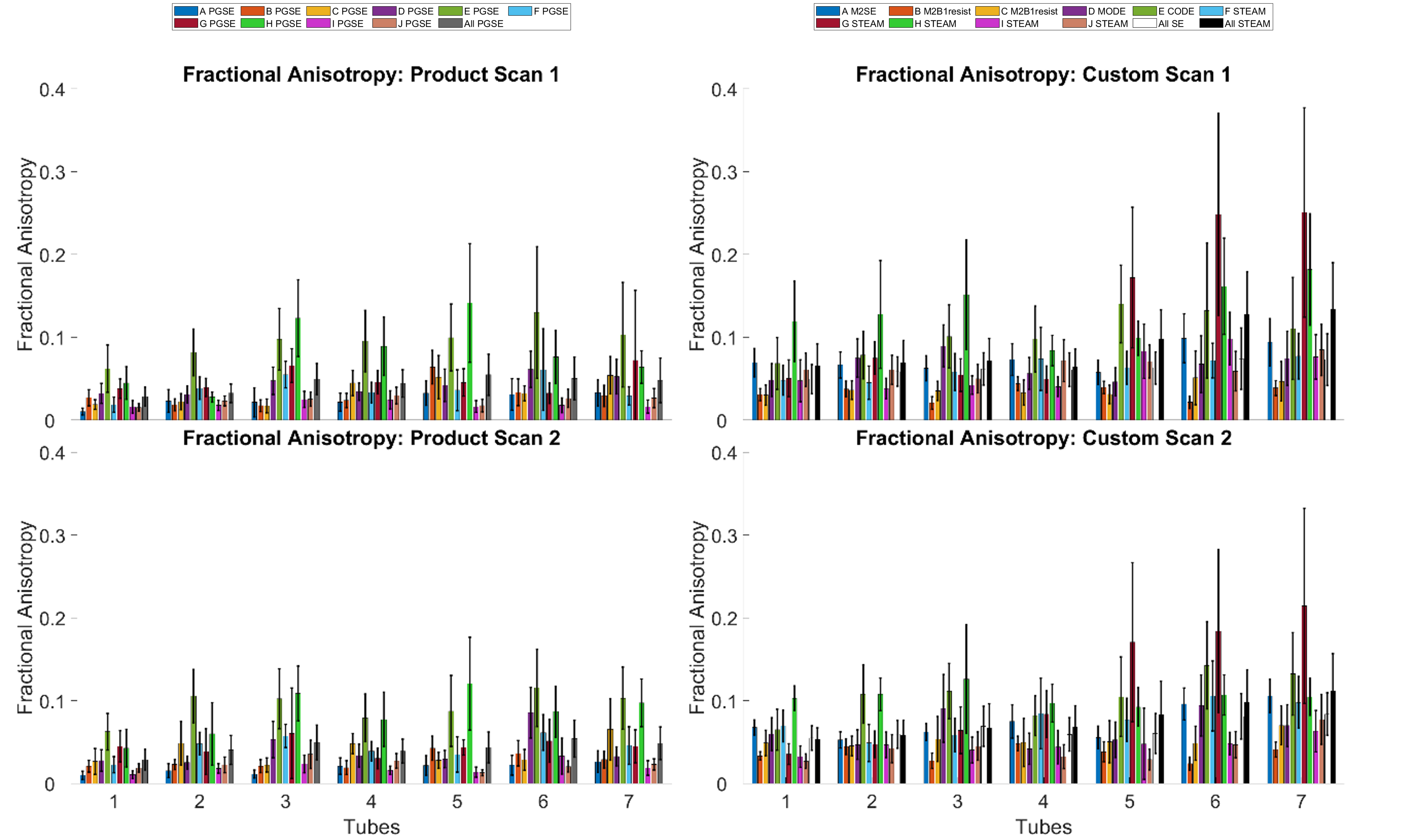

Supplement: Supplementary file 4 — Figure S4. Average FA across ROIs as a function of PVP concentration. Tensors were reconstructed using b = (100,450) s/mm2 data. Tubes 1–7 corresponded to (0, 2.5, 5, 7.5, 10, 15, 20) % PVP respectively. The expected value of FA in isotropic media is zero. An increasing trend in FA is observed with increasing PVP concentration. [file NBM-35-0-s002.tif]
