# Supplementary material for: Validation of cardiac diffusion tensor imaging sequences: A multicentre test–retest phantom study
Source: NMR Biomed. 2022 Feb 8;35(6):e4685. doi: 10.1002/nbm.4685 (PMC9285553; doi:10.1002/nbm.4685)
Supplement: Supplementary file 5 — Table S1. Mean difference between Scans 1 and 2, averaged across all tubes. [file NBM-35-0-s006.docx]

Supplementary Table 1. Mean difference between Scans 1 and 2, averaged across all tubes.

| **Parameter** | **Sequence** | **Site** | | | | | | | | | |
| --- | --- | --- | --- | --- | --- | --- | --- | --- | --- | --- | --- |
|  |  | **A** | **B** | **C** | **D** | **E** | **F** | **G** | **H** | **I** | **J** |
| MD (× 10^-5^ mm^2^/s) | Product | 1.02 | 0.39 | 1.27 | -0.36 | 2.04 | 0.20 | 0.25 | 0.64 | -0.26 | 1.02 |
|  | Custom | 0.24 | 1.21 | 0.61 | -0.68 | 3.41 | -0.29 | 0.56 | 1.00 | -0.68 | -0.31 |
| FA (× 10^-2^) | Product | -0.63 | -0.29 | 0.39 | -0.18 | -0.19 | 0.56 | -0.34 | 0.40 | 0.01 | -0.04 |
|  | Custom | -0.09 | 0.36 | 1.48 | -0.01 | 0.26 | 1.50 | -1.42 | -2.65 | -1.47 | -2.24 |

PGSE is denoted by product sequence, and SE (Sites A-E) and STEAM (Sites F-J) are denoted by custom sequence.
