# Supplementary material for: Validation of cardiac diffusion tensor imaging sequences: A multicentre test–retest phantom study
Source: NMR Biomed. 2022 Feb 8;35(6):e4685. doi: 10.1002/nbm.4685 (PMC9285553; doi:10.1002/nbm.4685)
Supplement: Supplementary file 6 — Data S1. Supporting Information [file NBM-35-0-s004.docx]

**SUPPORTING INFORMATION**

Includes *Data Acquisition*, *Stability of Measurements, Fractional Anisotropy, Supplementary Table 1 and Supplementary Figures 1 - 4.*

Data Acquisition

Each site performed imaging with one custom sequence of their preference. These included SE and STEAM-based approaches, including variants of spin echo with different motion-compensated diffusion waveforms including M2SE, CODE, MODE and M2B1resist. Due to heterogeneity in custom sequences, it was not feasible to standardise all sequence parameters without compromising the performance of other custom sequences. For example, STEAM has a shorter minimum echo time than SE, and SE generally requires higher gradient performance than STEAM. To avoid bias towards any given sequence, sites were asked to optimise their preferred methods whilst maintaining basic parameters including FOV, resolution, orientation and acquisition time used in the product sequence.

Stability of Measurements

To investigate the stability of measurements, data were reconstructed from single repetitions of DTI data, and the results shown in Supplementary Figure 1. Spikes were seen in the PGSE MD (Site G) and FA (Site J) data. The average drift in MD across repetitions and both scans was -0.06%, +0.03% and +0.43% for PGSE, SE and STEAM respectively, with maxima of +1.2% (Site J product sequence Scan 1) and -1.7% (Site G product sequence Scan 2). The average drift in FA across repetitions and both scans was +2.4%, +1.1% and +1.2% for PGSE, SE and STEAM respectively, with maxima of +40.2% (Site G product sequence Scan 1) and -28.8% (Site J product sequence Scan 1). The MD reconstructed from single repetitions was similar to that reconstructed from all repetitions. However, the FA reconstructed from single repetitions and averaged across repetitions and Scans 1 and 2 was elevated at 0.04, 0.06 and 0.13 in the PGSE, SE and STEAM data.

The SD across repetitions is illustrated in Supplementary Figure 2. In Tube 1, SD_MD_ across both scans were (1.4, 1.3, 5.3) × 10^-5^ mm^2^/s in PGSE, SE and STEAM, which corresponded to CV_MD_ of 1.2%, 1.1% and 4.7% respectively. The corresponding SD_FA_ were 0.012, 0.012 and 0.041. While SD_MD_ remain relatively stable across PVP concentrations, there was an increasing trend in SD_FA_ with PVP concentration in the STEAM data.

We investigated the stability of the DTI data across repetitions and found that the largest drift occurred during the product sequence (Sites G and J). For consistency, the protocol specified that the product sequences be run prior to the custom sequences. This may have led to inconsistent heating rates across scanners depending on prior scanner usage. To mitigate this effect, ‘warm-up’ scans could be specified to heat up the scanner hardware to a more consistent state. The CV of MD across repetitions were relatively low in PGSE and SE, indicating stable measurements of MD reconstructed from single repetitions. The CV_MD_ in the STEAM data were 327% higher respectively than that of SE. Moreover, the average FA reconstructed with single repetitions and averaged across repetitions was 126% higher in the STEAM data compared to the SE data. This points to lower SNR in the single repetition STEAM data that could cause a positive bias in FA or image artefacts varying between repetitions.

Fractional Anisotropy

A similar dependence in stability of FA on b-value combinations compared to MD was observed where FA = 0.035 ± 0.026, 0.059 ± 0.030, 0.11 ± 0.10 across b-value combinations in PGSE, SE and STEAM, with STEAM showing the greatest dependence on choice of b-values (Supplementary Figure 3). The RMSD of FA between Scans 1 and 2 in the custom sequence data were (0.076, 0.025, 0.018, 0.023, 0.017, 0.016, 0.017) across the b-value combinations b_low_,100; b_low_,300; b_low_,450; 100,300; 100,450; 300,450; b_low_,100, 300, 450 respectively.

Based on the combination of b = 100 and 450 s/mm^2^, the corresponding average FA were (0.028, 0.052, 0.059). Relative to the expected value of FA in isotropic media of zero, STEAM yielded the most elevated result, followed by SE and PGSE (Supplementary Figure 4). There was substantial variation in custom sequence data between sites, with average FA in Tube 1 across scans 1 and 2, ranging from 0.03 (Site B) to 0.11 (Site H). In tubes away from isocentre, the overestimation of FA was enhanced considerably. In Tube 7, the average FA for STEAM and SE were 0.13 and 0.078 as compared to 0.048 for PGSE. The standard deviation across ROIs in Tubes 1-7 is described as the regional heterogeneity. In Tube 1, the regional heterogeneity in FA ranged from 0.006 (Site B) to 0.032 (Site H). The average regional heterogeneity in FA across all sites and both scans were (0.012, 0.016, 0.020) for PGSE, SE and STEAM respectively. Regional heterogeneity increased with PVP concentration, particularly in the STEAM data.

**SUPPLEMENTARY FIGURE LEGEND**

Supplementary Figure 1. Intra-scan stability of MD and FA across repetitions.

Time course of MD and FA in Tube 1 (0% PVP) reconstructed from single repetitions of b = (100,450) s/mm^2^ with 6 DW directions. MD measurements were generally stable over repetitions with drift < 0.5% as averaged across PGSE, SE and STEAM data. The drift in FA across repetitions was larger, averaging +2.4%, +1.1% and +1.2% across PGSE, SE and STEAM data. Considerable FA drift was seen in specific sites and in the product sequence data, where |drift(FA)| > 10% in 4 sites using the product sequence, and none using the custom sequences.

Supplementary Figure 2. Intra-scan stability of MD and FA.

Values expressed as standard deviation across repetitions. SD_MD_ and SD_FA_ were highest in the STEAM data, and this effect was more pronounced at higher PVP concentrations.

Supplementary Figure 3. Sensitivity of FA to b-values used in the DTI reconstruction.

Average FA in Tube 1 (0% PVP) across sites, 2 timepoints, sequences and b-value combinations are shown. The two timepoints are denoted by the numerical suffix in the figure legends; product (left) and custom sequence data (right) are shown. Average values for PGSE, SE and STEAM are given by grey, white and black bars respectively. The b-values of non-DW data are denoted by b_low_, and ranged from 0 to 76 s/mm^2^ across sites (Table 1).

Supplementary Figure 4. Average FA across ROIs as a function of PVP concentration.

Tensors were reconstructed using b = (100,450) s/mm^2^ data. Tubes 1-7 corresponded to (0, 2.5, 5, 7.5, 10, 15, 20) % PVP respectively. The expected value of FA in isotropic media is zero. An increasing trend in FA is observed with increasing PVP concentration.
